# Substrate and cell fusion influence on slime mold network dynamics

**DOI:** 10.1038/s41598-020-80320-2

**Published:** 2021-01-15

**Authors:** Fernando Patino-Ramirez, Chloé Arson, Audrey Dussutour

**Affiliations:** 1 School of Civil and Environmental Engineering, Georgia Institute of Technology, Atlanta, GA 30309 USA; 2 Research Centre on Animal Cognition (CRCA), Centre for Integrative Biology (CBI), Toulouse University, CNRS, UPS, Toulouse, France

**Keywords:** Time-lapse imaging, Amoeboid migration, Chemotaxis, Dynamic networks, Robustness, Single-cell imaging, Time series

## Abstract

The acellular slime mold *Physarum polycephalum* provides an excellent model to study network formation, as its network is remodelled constantly in response to mass gain/loss and environmental conditions. How slime molds networks are built and fuse to allow for efficient exploration and adaptation to environmental conditions is still not fully understood. Here, we characterize the network organization of slime molds exploring homogeneous neutral, nutritive and adverse environments. We developed a fully automated image analysis method to extract the network topology and followed the slime molds before and after fusion. Our results show that: (1) slime molds build sparse networks with thin veins in a neutral environment and more compact networks with thicker veins in a nutritive or adverse environment; (2) slime molds construct long, efficient and resilient networks in neutral and adverse environments, whereas in nutritive environments, they build shorter and more centralized networks; and (3) slime molds fuse rapidly and establish multiple connections with their clone-mates in a neutral environment, whereas they display a late fusion with fewer connections in an adverse environment. Our study demonstrates that slime mold networks evolve continuously via pruning and reinforcement, adapting to different environmental conditions.

## Introduction

Transportation networks where fluids are transported from one point of the network to another are ubiquitous in nature. Vascular networks in animals, plants, fungi and slime molds are commonly cited examples of such natural transportation networks. These networks are often studied as static architectures, although most of them have the ability to alter their morphology in space and time in response to environmental conditions^1^. Morphological alterations often include short term changes in the vein diameter and long-term structural adaptation such as addition or loss of veins^1,2^. The slime mold *Physarum polycephalum* is a unicellular organism that is often used to study problem-solving in single-celled organisms^3–8^ and constitutes an ideal model system to study short and long term alterations in network morphology, in response to environmental conditions.

The motion and behaviour of *Physarum polycephalum* rely on a complex internal architecture which consists of a complex network of interconnected veins. The main function of this vein network is to transport oxygen and nutrients to maintain homeostasis in cells that range from 10 square micrometres to 10 square meters^9^. These veins contract and relax periodically, causing the cytoplasm to flow back and forth—a phenomenon called “shuttle streaming”. These contractions produce a pressure gradient that pushes the cytoplasm towards the cell periphery where veins cannot be distinguished due to their high space concentration. The resulting pseudopods have a fan-like structure. The slime mold membrane extends and retracts in synchrony with the shuttle streaming, allowing cell migration at a speed up to few centimeters per hour^10^. In a homogeneous environment, the exploration process of slime mold alternates between refinement and generation of pseudopods that explore the domain^11^.

The frequency and the amplitude of the vein contractions depend on external cues^12^. For instance, when *Physarum polycephalum* perceives a localized chemical attractant such as glucose in the environment, the veins contract at a high rate and the membrane migrates toward the attractant^13–15^, whereas when it senses a localized chemical repellent such as Sodium Chloride (NaCl), the veins contract at a lower rate and the membrane retracts, moving away from the repellent^16^. It has been shown that slime molds growing in an environment where NaCl is homogeneously distributed slow down the exploration process to allow production of mucus protection, whereas slime molds that grow in an environment where glucose is homogeneously distributed slow down the exploration process to allow metabolization^11^. Hence, *Physarum polycephalum* can adjust its behaviour, shape, size and migration speed based on environmental conditions. Interestingly, as the slime mold moves and expands, its networks of veins are also evolving and growing^17^. A question that arises is how external cues affect network morphology.

*Physarum polycephalum* cells have been modeled as undirected graphs by a number of authors, which even resulted in the creation of a public repository of slime mold extracted graphs^18^, and different codes for network extraction from raw images^19,20^, which describe different segmentation networks based on the characteristics of the acquired images. Different studies on the network dynamics of slime molds suggest that the networks are hierarchical, with the distribution of veins widths and lengths following exponential, gamma or log-normal distributions, with the majority of the veins being short and thin, with a few long and thick veins^21–23^. As is the case in biological networks, the mean node degree is close to three in *Physarum polycephalum* networks, with little variations due to the existence of end branches. Thicker and longer veins have higher centrality than their adjacent veins^20,24^. Smaller, less central veins allow adaptability and redundancy of the network as it constantly evolves and coarsens as a result of exploration^20,23^. Even though the majority of studies on the network dynamics of *Physarum polycephalum* have focused on neutral environments, a recent study^25^ revealed that changes in vein diameters can be induced by localized chemical attractants. However, the impact of these changes on the complete network morphology was not investigated in detail. Takamatsu et al.^26^ and Ito et al.^22^ studied the effect of nutrients (oat flakes) and a mild repellent (KCl) on the network formed by *Physarum polycephalum*, showing that substrates containing oat usually yield networks with thinner veins but longer overall networks than substrates containing KCl, which promote thicker veins but shorter networks.

Another interesting feature of *Physarum polycephalum* is that it can be severed into viable and structurally similar yet smaller cells. Upon contact, these cells can fuse with each other. Slime mold fusion constitutes a defining feature of the lifestyle of slime molds , allowing them to share information once merged^27^, and even before it, through the layer of mucus deposited during growth^28^. When two different networks merge, remodeling might involve changes in vein diameter as well as pruning of supernumerary veins as seen in animal vascular systems^29^. Formation of an extended network by fusion of microplasmodia (micrometer-sized cell without tubular vein network) has been investigated^30^, but to our knowledge, the reorganization of *Physarum polycephalum* networks after fusion of macroplasmodia (millimeter-sized cell with a tubular vein network) has never been studied.

Hence, due to its extremely original behaviour, fast migration rate and outstanding network topology, the acellular slime mold *Physarum polycephalum* offers an attractive model for the analysis of morphogenesis dynamics underlying cellular migration, exploration and fusion. In this paper, we grow two slime mold cells of the same strain in adverse, nutritive and neutral environments in order to characterize the morphology, network and dynamics of *Physarum polycephalum* before and after fusion. First, we investigate whether and how the topology of slime mold networks is affected by different environmental conditions, by comparing an adverse environment (using sodium chloride NaCl as a repellent), a nutritive environment (using glucose as a chemo-attractant) and a neutral environment (using plain agar). Second, we analyze the evolution of slime mold networks within the 3 h after fusion. We develop a program that automatically analyzes sequences of images to track the area and shape of the surface covered and explored by the slime mold, transforms images of slime mold into undirected graphs, and calculates network cost, efficiency and resiliency indexes. Our program allowed us to run multiple experiments simultaneously, imaging up to 20 dishes at the same time, without affecting the precision of the computed cost and efficiency of the complete networks.

## Methods

### Species

The slime mold *Physarum polycephalum* is a unicellular organism that belongs to the Amoebozoa. Its vegetative state, the plasmodium, is a giant mobile cell that consists of a syncytium of nuclei and an intracellular cytoskeleton, which forms a complex cytoplasmic network of veins. Its cytoplasm consists of a viscous phase (ectoplasm) and a liquid phase (endoplasm) characterized by different concentrations of fibrous proteins. The ectoplasm, which contains actin and myosin, forms the contractile walls of the veins. Within these veins flows the endoplasm, which contains organelles such as nuclei and mitochondria. Ecto- and endo-plasms are convertible into one another. A starving plasmodium can enter a dormant stage, called sclerotium, and turn back to a plasmodium after being transferred to a fresh food medium. In this paper, we used the Australian strain provided by Southern Biological, Victoria, Australia. We revived a total of 12 sclerotia to conduct the whole study.

### Rearing conditions

The slime molds were reared on a 1% agar medium with rolled oat flakes. They were fed every day and their agar medium was replaced daily. Slime molds were 2 weeks old when the experiment started. All experiments were carried out in the dark, at a temperature of 25 degrees Celsius, a humidity of 80%, for 48 h. Pictures were taken with a Canon 70D digital camera. A light source underneath the petri dishes was turned on for 3 s when photographs were taken.

### Experimental setup

To investigate how the substrate affects the vein network evolution before and after fusion, we observed the behaviour of two slime molds that explore a medium that is neutral, slightly nutritive or adverse. Two circular slime molds (13 mm diameter) were placed directly on two opposite sides of a circular arena that consisted of a 90 mm diameter petri dish filled with either a plain 1% agar (neutral environment, i.e. control treatment), a plain 1% agar mixed with glucose (100 mM, nutritive environment), or a plain 1% agar mixed with salt (100 mM, adverse environment). It is important to note that while we called the substrate containing glucose “nutritive” as glucose in a nutrient, this substrate cannot sustain the slime mold growth by itself^31^. Hence, the slime mold biomass did not change in the course of the experiment. All slime molds were fed just before the experiment, so we assumed that they were in the same physiological state. We replicated the experiment at least 40 times for each substrate. We tested and monitored each arena for 48 h taking time-lapse photographs every minute. A LED panel underneath the petri dishes was turned on for 3 s when photographs were taken using a self programmed Arduino connected to the camera.

### Image segmentation

The image analysis algorithm was implemented in Matlab^32^. First, we masked manually the area outside of the contour of the petri dish. Then, a Laplacian filter was applied to enhance the contrast of the images. A traditional segmentation using the RGB space did not yield good results because the color region of slime mold (light yellow) was too similar to the color values of the background (white). Therefore, we proposed a segmentation method based on the LAB color space. Specifically, we used the components L (light) and B (blue to green), since the component A proved to be non-informative to the segmentation. The segmentation method in the LB space can be enhanced by adding a third dimension that is equal to the norm of the pixel-wise subtraction between the current image and the first image of the experiment (D). Then, the proposed LBD space could be segmented using any common algorithm, such as the k-means algorithm. Nonetheless, for the present study, we found that similar segmentation results could be found using a simpler approach: using the LB space, and reducing the image to an intensity array by computing L-B and normalizing the result to the interval [0,1]. Advantages were that the run-time of the algorithm was shorter, and that there was no need to broadcast the initial image of the test (to compute D) to every processor when using parallel computing. After the segmentation stage, the intensity image was binarized using the adaptive method for dark foregrounds proposed by Bradley and collaborators^33^.

**Figure 1:**
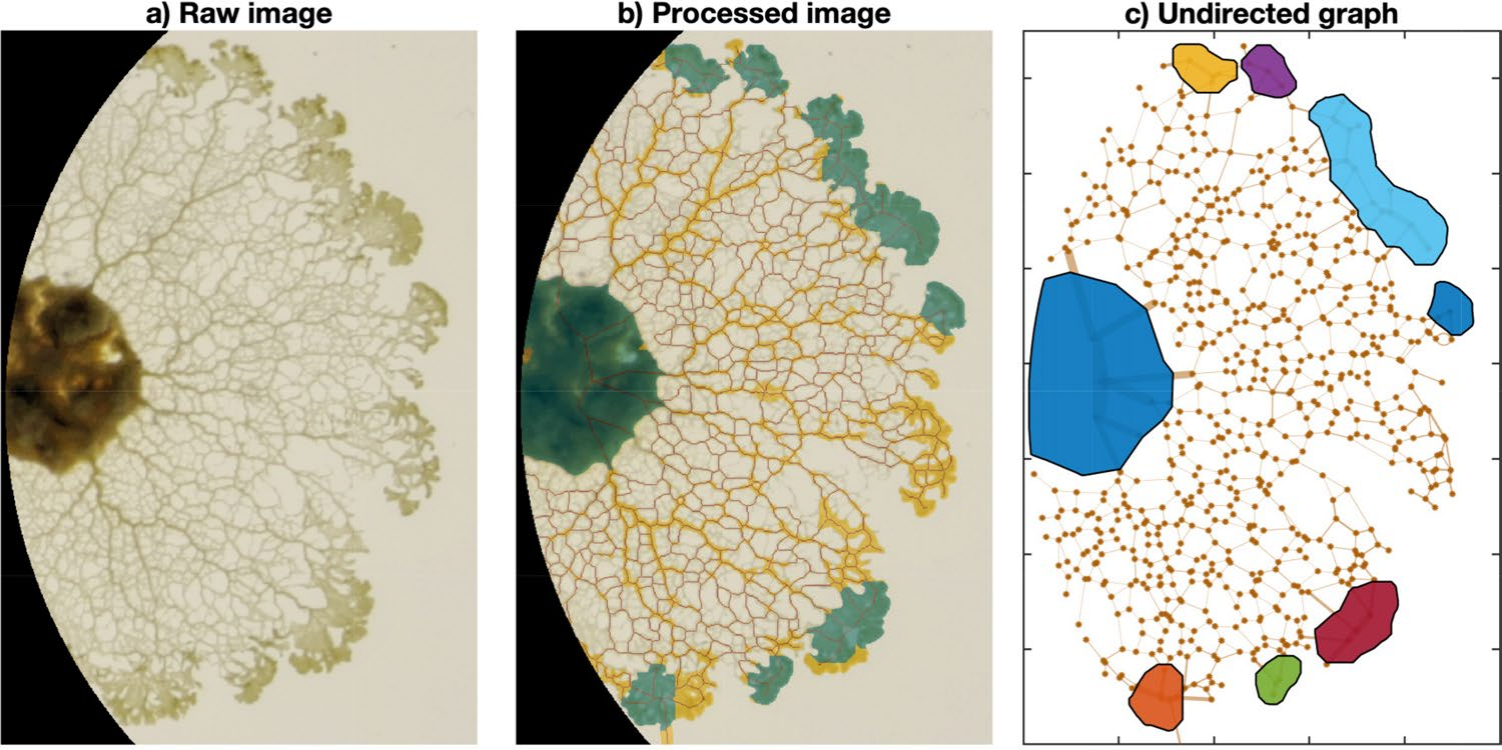
Segmentation process. (**a**) Raw image, masked at the boundary of the petri dish and enhanced used a Laplacian filter. (**b**) Segmented image overlay on original image, with highlighted pseudopods and cell topological skeleton. (**c**) Resulting undirected graph: dots and segments correspond to nodes and edges respectively. Approximate location of clusters shown as colored polygons. Three videos exemplifying the time-lapse segmentation process are available as supplementary material of this study. Figures generated using Matlab 2019a (https://www.mathworks.com/products/matlab.html).

Nonetheless, fading thin veins could not be captured by the binarization process. This limitation did not affect considerably the overall area calculation of the slime mold cell, but it impaired the calculation of connectivity parameters. Therefore, we coupled the binarization process with an edge detection subroutine that blurred the image by using a Gaussian filter ($\sigma =2$) and then performed ridge detection on the resulting array using a watershed procedure^34^. This subroutine yields an image that better identified thin veins but not large slime mold regions. The union of the segmentation and ridge-detection arrays gave a binary image that combined the benefits of both methods, and was then used in the subsequent steps. A sample segmented image is shown in Fig. 1. In addition, three videos (one per substrate type) are shown in the supplementary materials.

### Morphological and graph network indexes—before fusion

In the present study, we analyzed the influence of different substrates on the evolution of the slime mold network, initially focusing on each individual slime mold and later studying the network dynamics at the location where the slime molds fuse. In both studies, morphological an topological indexes were computed in order to quantify the observations. Moreover, in order to reduce the time needed for the program to automatically find the fusion time, we provided an initial guess based on visual inspection. The algorithm could function without this initial guess, which is optional. If an initial guess is provided, the algorithm refines the solution by finding the earliest image in which the two slime molds became connected to form a single object. If an initial guess is not provided, the program iterates from a random guess.

#### Morphological indexes

The pre-fusion analysis was performed by comparing networks extracted after the slime mold cell had covered a certain area (Covered Area CA) relative to the initial slime mold area (circle of 13mm in diameter). In total, we compared networks for seven normalized areas, from 1 to 4 times the initial area of slime mold, at 0.5 intervals. The time at which the slime molds reached a given normalized area was found by using linear interpolation between known time-area pairs. Initially, the only known areas were the initial area ($t=0$) and the area at the fusion time. In the fusion analysis, we compared networks from the time of fusion, up to 3 h afterwards.

Besides the slime mold cell area (CA), measured from the binary image, the so-called print area (PA) was calculated as the total area enclosed by the cell contour, e.g. including the empty regions inside it. The number of empty regions inside the slime mold cell was computed as well, together with the total area covered by these regions (PA–CA), which corresponds to the empty space inside the slime mold cell. The ratio of empty space to slime mold area (CA) has a value of 0 for a slime mold cell with no empty regions or “holes”, and increases with the empty space area. The mean size of the empty regions was calculated as the ratio between the total empty area and the number of such regions.

The total length of the network was found from the topological skeleton of the segmented image, also known as medial axis, following Matlab’s implementation of the algorithm proposed by Lee and collaborators^35^, as highlighted in red in Fig. 1, panel b. The average vein width was then approximated as the ratio between the slime mold area and the total length of the network. In addition, the algorithm proposed by Maurer^36^ allowed us to calculate the Euclidean distance transform along the skeleton, which is the distance from the skeleton (medial axis) to the closest edge of the slime mold cell, and therefore corresponds to half the vein width. The pseudopodia of the slime mold cell were identified by sequentially eroding and dilating the image with a structuring element of 1*mm* in radius. The remaining regions after such an operation were then labeled as pseudopods (shown in green in Fig. 1. The proportion of pseudopods in the networks is defined as the ratio between the area of the pseudopods regions and the total slime mold area.

#### Undirected graph indexes

From the binary image, we obtained the topological skeleton and the distance transform along that skeleton. From those, we constructed undirected graphs to represent the slime mold networks. In these graphs, the nodes correspond to the branch points and the end points of the skeleton, while the segments between them correspond to the edges. Each edge is associated to its parent nodes. The edge width is equal to the average value of the vein widths along it, which was calculated as two times the distance transform value. We also calculated the length of each edge, the Euclidean distance between the parent nodes, and the drag of the edge. The drag can be thought of as the resistance to flow: it was calculated as the ratio between the edge length and the fourth power of the edge width.

**Figure 2:**
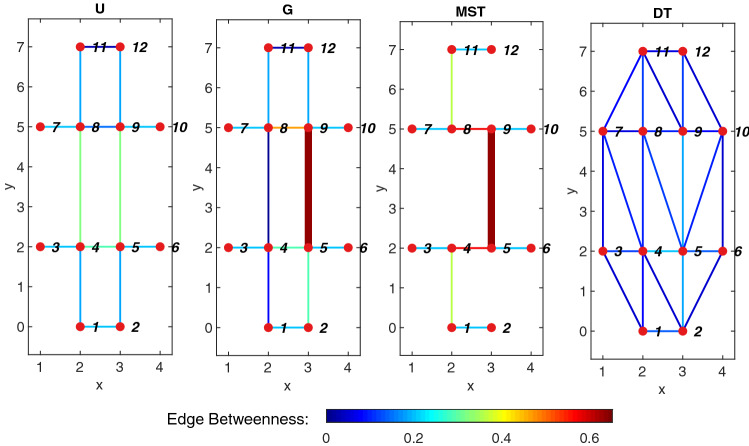
Sample graph. U: sample undirected graph connecting 12 nodes, all the edges in the graph have a uniform edge width. G: sample graph with the same topology as graph U, but an increased edge width between nodes 5 and 9. MST: Minimum spanning tree for U and G, some edges disappear from the graphs to form a simply connected network with minimum length. DT: Delaunay triangulation of graphs U and G, the triangulation is a maximum connected graph between the nodes, irrespective of the edge or node weights of the graph. The edge color is proportional to the edge betweenness in the graph.

We stored the coordinates of each node and we assigned a type to each node, depending on whether the node fell inside a pseudopod, the initial cell, or the veins. After constructing the graph, a subroutine calculated the connectivity of each node, and simplified the graph in order to avoid self-loops and repeated edges between nodes. A sample undirected graph constructed from a raw image is shown in Fig. 1, panel c. Four different categories of graph indexes were computed: connectivity, cost, transport efficiency and resiliency/hierarchy, as explained below. The indexes are illustrated in the simple sample graph shown in Fig. 2.

#### Connectivity

The node degree corresponds to the number of edges connected to a given node. It is a common metric used to quantify the “connectedness” of a network. Nevertheless, biological networks tend to form degree 3 networks with little variations, and therefore the mean node degree did not provide much information on network connectivity. The alpha index^37^ is a measure of the density of cycles in the graph (also named loops), ranging from 0 for simply connected networks (no loops) to 1 for maximum planar networks, which are networks in which no extra edges can be added without crossing existing ones (e.g. Delaunay triangulations). The alpha index was computed but, similar to the connectedness index, results were non-informative.

#### Network cost

We quantified the network cost in terms of the weighted wiring cost under different scenarios. The formal definition of the wiring cost is the sum of the upper (or lower) triangular weighted adjacency matrix of the graph. If the edge weight corresponds to the length of the edges, the wiring cost corresponds to the total length of the network. Instead, if the weight metric is the Euclidean distance between connected nodes, the wiring cost corresponds to the total length of a network with the same topology, but with strictly straight segments between nodes. Then, we defined the tortuosity of the network as the ratio between the total network length (wiring cost by length) and the length of the equivalent graph with straight edges (wiring cost by Euclidean distance).

Furthermore, we normalized the total network length by comparing it to its upper and lower bounds. The minimum spanning tree (MST) is the subset of edges of the original graph that connects all the nodes with minimum total edge weight, and therefore is a lower bound for network cost. For instance, the MST in terms of length is the third graph in Fig. 2. In this example, the MST of U in terms of length or drag is not unique, given that the graph has double symmetry and that there is more than one solution that gives the minimum network length/drag. For instance, choosing either edge from 4–8 or 5–9 will yield the same (minimum) network length/drag. On the contrary, the MST in terms of drag for graph G strictly selects edge 5–9 over edge 4–8.

The upper bound for network cost is the Delaunay triangulation (DT), which is the maximally connected graph for the set of nodes in the graph, irrespective of weight measures. A maximally connected graph is such that there is no way to add an extra edge to the network without crossing any other existing edge. Given that the DT of a graph depends only on the geometric distribution of its nodes, not the weight of the edges, the triangulation (DT) shown in Fig. 2 is the same for graphs U and G. Once we found the comparison graphs for each network, we mapped the network cost to the interval [0, 1] where zero and one correspond to the lower and upper bounds, respectively.

#### Transport efficiency

Transport efficiency was quantified based on the travel distance (shortest path) and the drag between source and sinks. Similar to the network cost, we compared the transport efficiency between pairs of nodes in the network against a lower bound. We computed the shortest path between every pair of nodes along the network (using Matlab’s implementation of Dijkstra’s algorithm) in terms of edge length, and its lower bound, the Euclidean distance between node coordinates. Then, we calculated the length efficiency index (LE) defined^38^ as follows:

1 $$\begin{aligned} LE = \frac{\sum _{ij}^{}l_{ij}^{-1}}{\sum _{ij}^{}e_{ij}^{-1}} \end{aligned}$$

where $l_{ij}$ and $e_{ij}$ are the path length and the Euclidean distance between nodes *i* and *j*, respectively.

Referring to the sample graphs in Fig. 2, one can see the differences in transport efficiency, for instance between nodes 2 and 7 of the graphs. It becomes evident that the DT offers the best efficiency, since the path from node 2 to 7 passes only through nodes 2, 4 and 7, with a total path length that is similar to a straight line between nodes 2 and 7. Conversely, the same path along graph U passes through nodes 2, 1, 4, 8 and 7 (it is not unique), with a path length that is clearly longer than a straight line, and therefore, with lower efficiency. Moreover, the same path along the MST passes through nodes 2, 1, 4, 5, 9 and 8, yielding an even longer path length and lower efficiency, showing that network cost and path efficiency are oftentimes inversely correlated.

In addition to the general efficiency index (LE), we also studied the distribution of path efficiency, by computing $e_{ij}/l_{ij}$ for each pair of nodes *i*, *j* and by calculating the mean value and the coefficient of variation (COV, standard deviation divided by the mean) of the set of individual path efficiencies.

Similarly, we computed the pairwise shortest paths in the graph, this time in terms of minimum drag between nodes $d_{ij}$. In this case, the lower bound is a path with minimum possible drag between nodes, which corresponds to the shortest path (straight line) through an infinitely wide vein, which equals zero. For comparison, our lower bound $c_{ij}$ was the drag between nodes *i*, *j* through a vein with a diameter of 0.5 mm and shortest possible length (euclidean distance). Setting an arbitrary vein width (in this case 0.5 mm) can shift and stretch the values of drag efficiency, but it does not modify its distribution, and therefore, it does not alter the relative values among graphs. We thus computed the mean and COV of the drag efficiencies $d_{ij}/c_{ij}$ for every graph, and we compared the values relative to one another instead of discussing the significance of their magnitudes.

#### Edge hierarchy and network resiliency

In order to quantify the relative importance of edges in the graph, we calculated the edge betweenness centrality, defined as the percentage of all the shortest paths between nodes that pass through each edge. Similar to the shortest path efficiency, we calculated the edge betweenness in terms of length and drag. An example of the edge betwenness distribution is shown in Fig. 2 in the form of edge colors. From the DT, it is evident that each edge is used by approximately $8\%$ of the shortest paths between nodes, with a very homogeneous distribution. On the contrary, more centralized networks such as the MST, show the dependency of the network on some edges, such as edge 5–6 which is used in about $65\%$ of the shortest paths, since it is the only node connecting the upper and lower regions of the graph. A comparison between the drag betweenness of graphs U and G shows the influence of the edge weight (drag in this case), even though the topology of both networks is the same, the low drag of edge 5–9 in graph G skews the betweenness distribution, showing that this edge becomes significantly more important in the graph in comparison to edge 4–8. Apart from the difference in drag between those edges (graph U), the load is distributed and becomes symmetric at about $32\%$. The frequency distribution of betweenness for the graphs followed an exponential distribution for both metrics (length and drag); it was parametrized by the parameter $\mu$, equivalent to the mean of the edge betweenness values.

In addition, we studied the network resiliency in terms of the fault tolerance (FT) of the graphs^38–40^. The FT corresponds to the percentage of edges that can be removed from the graph while still connecting a given percentage of the nodes, usually $50\%$. The selection of the edges to remove was random and so the FT was calculated as the average of 30 independent realizations of the procedure. In order to provide bounds of comparison, we calculated the FT for the MST (lower bound) and the DT (upper bound) of the graph. We mapped the FT of the actual network to the interval [0,1] where the bounds correspond to the MST and the DT, respectively.

Moreover, in order to study the effect of edge hierarchy in the resiliency of the network, we proposed a modified procedure to calculate the FT, this time by systematically removing the *k* edges with the highest/lowest drag betweenness with $1\%\le k\le 100\%$ of the number of edges in the graph. Removing the most ’important’ edges of the graph (highest betweenness) is considered a worst-case scenario, and starting from the least important edge, a best-case scenario.

### Cell fusion indexes

We tracked the fusion region from fusion time (FT) up until 3 h after fusion, by analyzing images every 15 min. In order to find the fusion region, we compared the print of both slime mold cells right before fusion (BF), and the print of the fused slime mold 5 min after fusion (AF). The difference between AF and BF (AF-BF) gave an image of the newly grown regions ($r_i$), from which, one (or more) vein connected the two slime mold cells. We calculated the region obtained by adding each $r_i$ to BF until the regions became connected. Then, at FT, we extracted the region of slime mold that was within 5 mm from the connecting region. In order to capture possible expansion of slime mold adjacent to this region, we added an offset of 2 mm around its contour. The resulting area was defined as the fusion region (FR).

Then, at each time step, we measured the slime mold area, print area, ratio of empty and slime mold areas, average size of the enclosed empty regions, network length and mean and maximum vein widths inside the fusion region (FR). We observed large variability among the index values, attributed to the wide differences among the shape and extent of the fusion regions themselves. To alleviate this issue and better observe the evolution after fusion, most of these indexes were normalized by their initial value at fusion. Indexes specific to the fusion region were defined based on graph analysis, and are explained in the following section.

#### Fusion region graph indexes

**Figure 3:**
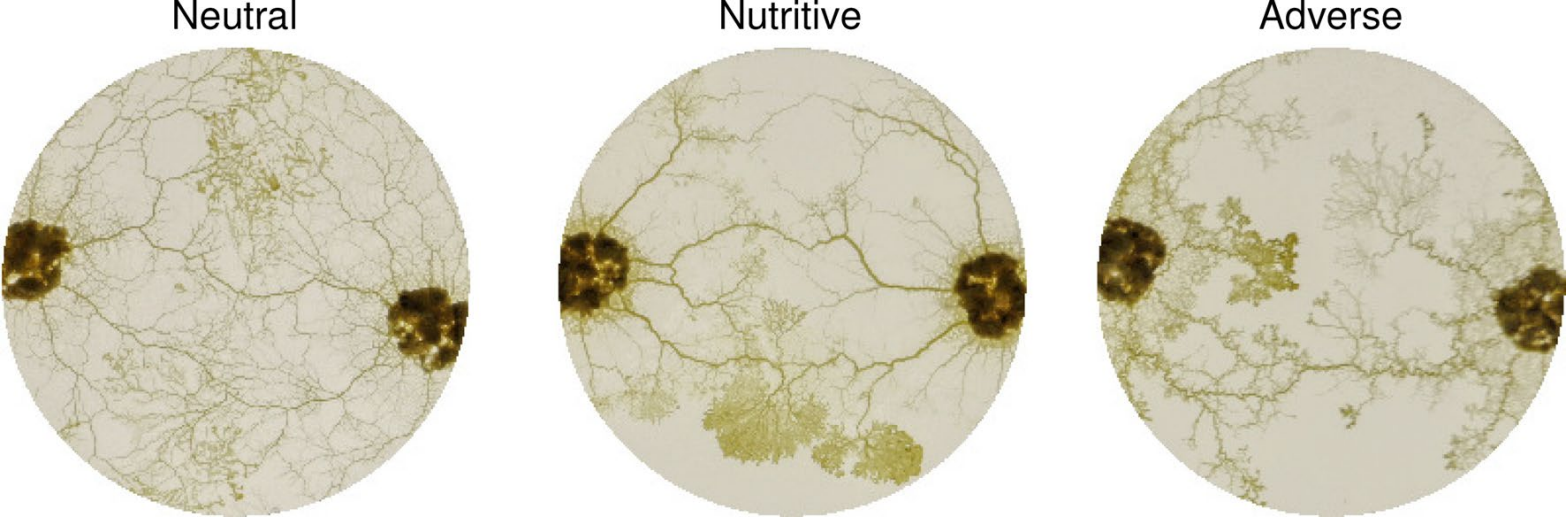
Fused slime mold cells. Raw images taken 3 h after initial fusion of the slime mold cells in different substrates. Images illustrate the influence of the substrate in the number and thickness of connecting veins between sides of the network.

We analyzed the evolution of the graph within the fusion region in terms of number of nodes and edges, mean and maximum edge thickness within FR. In addition, we studied the connectivity between the two initial slime mold cells and its evolution over time, which was highly variable among environments, as shown in Fig. 3. To do this, we calculated the shortest path between the two initial locations of the slime mold cells in terms of length and drag and in terms of the number of veins connecting the initial slime mold cells. The number of connections between the initial slime mold cells was calculated from the maximum flow of the graph, using Matlab’s implementation of Boykov-Kolmogorov’s algorithm. In general terms, the maximum flow of the graph between two nodes is the critical edge capacity that limits the flow between such nodes. Then, in the case of unweighted graphs (i.e. every edge has a weight of 1), the maximum flow corresponds to the maximum number of distinct paths between the nodes, i.e. the number of connecting veins.

Then, in order to count the maximum number of veins connecting initial slime mold cells, we used an initially unweighted graph, we picked one node inside each initial cell, and increased the weight of their surrounding edges to a large value. This to make sure that the connections bottleneck occurred in the connection between both sides of the network. Specifically, we increased the weight of the edges within 25 mm of each source node to a value of 100. Then, the maximum flow between the nodes corresponded to the number of connecting veins between the initial slime mold cells. Furthermore, following the same procedure with a graph weighted by its edge width, we calculated maximum flow as the total bandwidth between both sides of the graph. We present the results both in terms of number of connecting veins and in terms of the average width of the edges, which was calculated as the total bandwidth divided by the number of connecting veins. In a similar way, the shortest path between the initial slime mold cells was tracked over time, both in terms of minimum path length between initial slime mold cells and in terms of minimum drag path.

### Statistical analyses

To assess the difference in the various parameters measured between the three treatments, we used linear mixed models (function lmer, Package lme4) or generalized linear mixed models (function glmer, Package lme4^41^) in R (RStudio Version 1.2.1335). The models were fitted by specifying : the response variables (the various indexes defined above), the explanatory variables: treatment (categorical predictor), the normalized area or the time after fusion (continuous predictors) and the random effects: the plasmodium from which each individual slime molds were cut from (plasmodium identity) and the replicate (slime mold identity). The response variables that did not fit linear model requirements were transformed using the “bestNormalize” function (“bestNormalize” package^42^). The outcomes of all the models are presented in the supplementary information.

## Results

The following section is divided into two parts. First, we study the influence of the different environments (neutral, adverse and nutritive) on the network characteristics of the slime mold cells, growing freely before fusion. Secondly, we analyze the network dynamics after fusion in the different environments.

### Influence of substrate on slime mold networks

#### Slime mold morphology analysis

**Figure 4:**
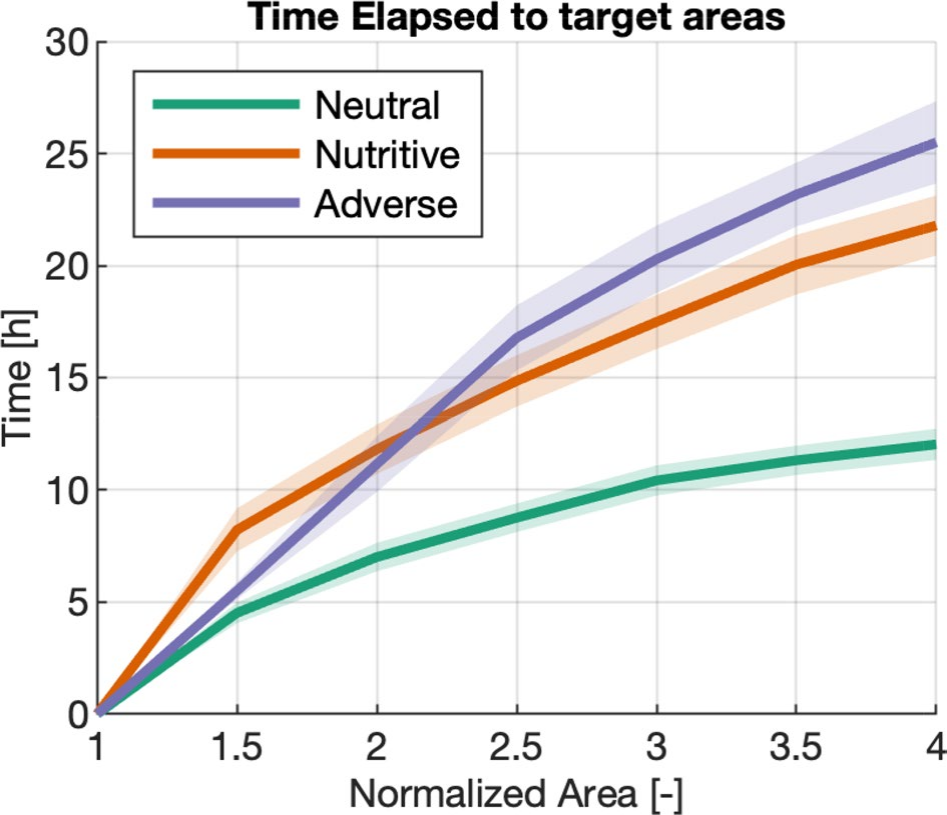
Time elapsed to target areas. Slime mold area normalized by the initial cell size (horizontal axis) versus time elapsed to reach such area (vertical axis). Solid lines correspond to the mean value among replicates for the same substrate and shaded regions correspond to the confidence interval.

The first step of the analysis focuses on the expansion rate of the slime mold cells in different environments, as shown in Fig. 4. Results show that slime molds exploring a neutral environment expand to cover four times their initial area after 10 h whereas slime molds exploring a nutritive or an adverse environment reach the same area only after 21 and 26 h in average, respectively (Table S1, Fig. 4). This result confirms that the expansion rate depends on the substrate characteristics, as observed in our previous study^11^.

**Figure 5:**
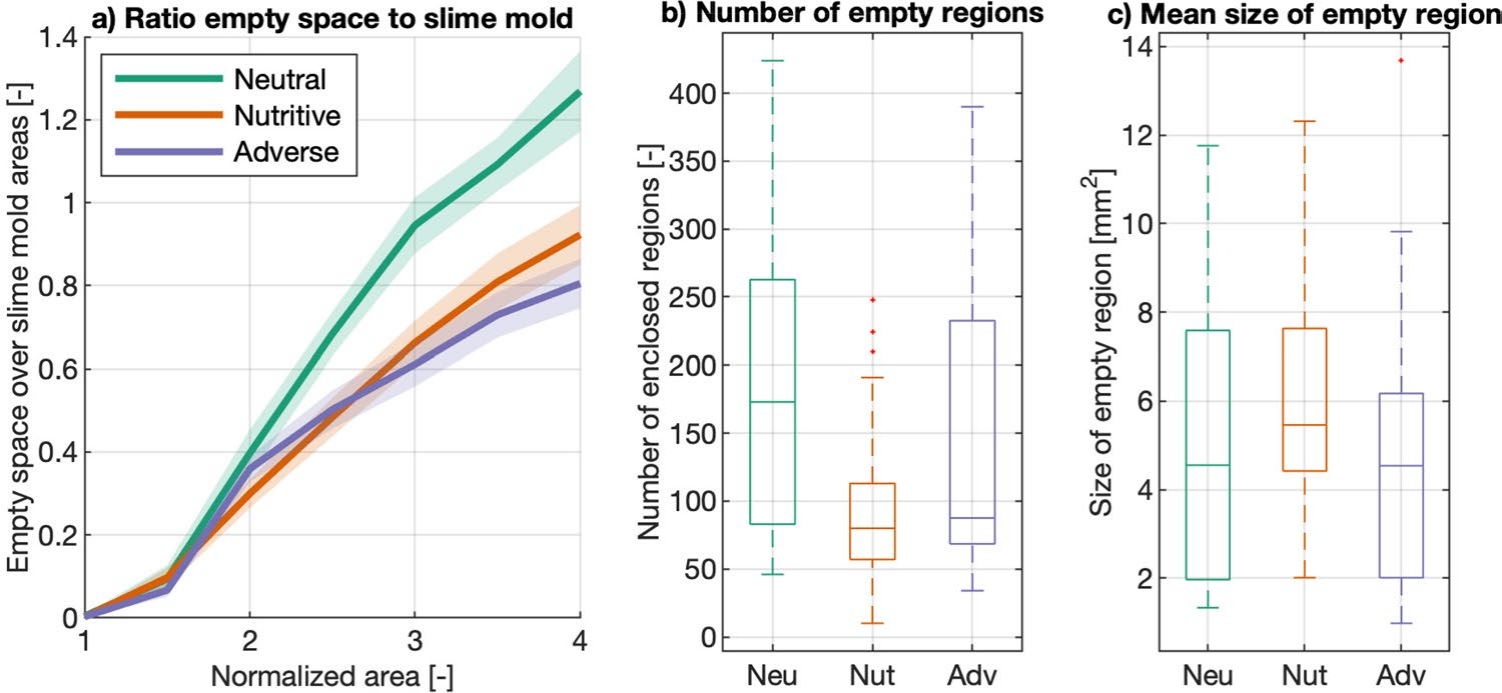
Empty regions inside slime mold cell. (**a**) Ratio between empty space inside slime mold cell and slime mold area as a function of normalized slime mold cell area (An). The solid lines and shaded areas correspond to the means and confidence intervals among replicates, respectively. (**b**) Boxplot graph of the number of enclosed empty regions inside the slime mold cell at An = 4. (**c**) Boxplot graph of the mean size of the empty region at An = 4, computed as the total empty area divided by the number of enclosed regions. Box plots show median (horizontal line), interquartile range (box), distance from upper and lower quartiles times 1.5 interquartile range (whiskers), and outliers ($>1.5x$ upper or lower quartile).

Note that the total area enclosed by the whole slime mold cells is not always the same. Therefore, we quantified the ratio between the empty area (not covered by slime mold) and the slime mold cell area, as shown in Fig. 5.

While expanding on a substrate, slime molds build networks that enclose empty regions and appear as mesh net structures. The total area covered by such empty regions is larger when a slime mold is exploring a neutral substrate than the other two substrates (Table S2, Fig. 5a). Since we are comparing the geometry and network of slime mold cells that cover the same area (CA), it also means that the total area explored by slime molds (print area PA) in the neutral environment is larger than on the other two substrates. The number of empty regions is the lowest on a nutritive substrate and the highest on the neutral substrate (Table S3, Fig. 5b) while the area of the empty regions is the smallest on the adverse substrate (Table S4, Fig. 5c). Thus, on a neutral substrate, the slime molds build sparse networks enclosing numerous empty spaces, while on a nutritive substrate, they construct networks presenting few but large empty regions, and on an adverse substrate, they establish a tight and compact network. We also notice that the proportion of pseudopods is the highest on a nutritive substrate (Tables S5–S6).

#### Slime mold network topology analysis

**Figure 6:**
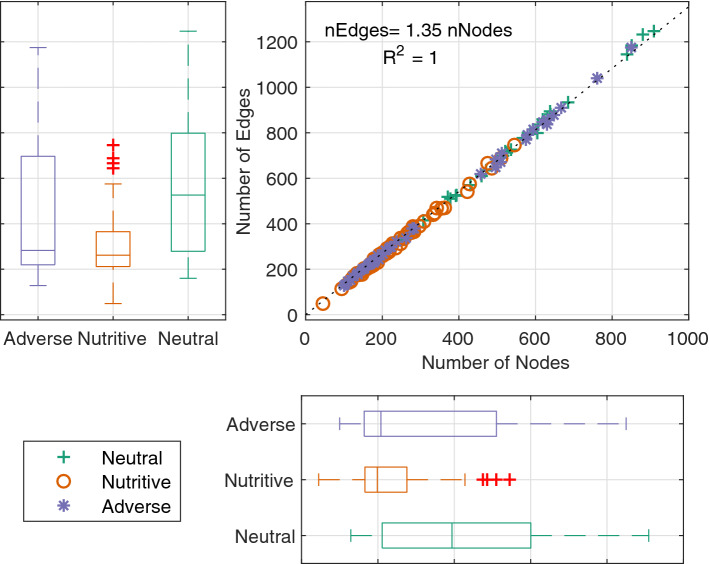
Number of nodes and edges in the network. Boxplots showing the variability of the number of Edges and Nodes in the networks by substrate. Scatter plot showing the linear relationship between the number of edges and nodes. Box plots show median (horizontal line), interquartile range (box), distance from upper and lower quartiles times 1.5 interquartile range (whiskers), and outliers ($>1.5x$ upper or lower quartile).

The topology analysis of the slime mold networks first shows that on all substrates, the number of edges and the number of nodes are highly correlated regardless of the network size. The mean node degree is in average 2.65. Figure 6 shows that there is a linear relationship between the number of nodes and edges in the networks, which holds for every environment and cell size studied. The fact that the number of edges is consistently $35\%$ more than the number of nodes explains that the connectivity of the networks is an inherent characteristic of the slime mold networks.

**Figure 7:**
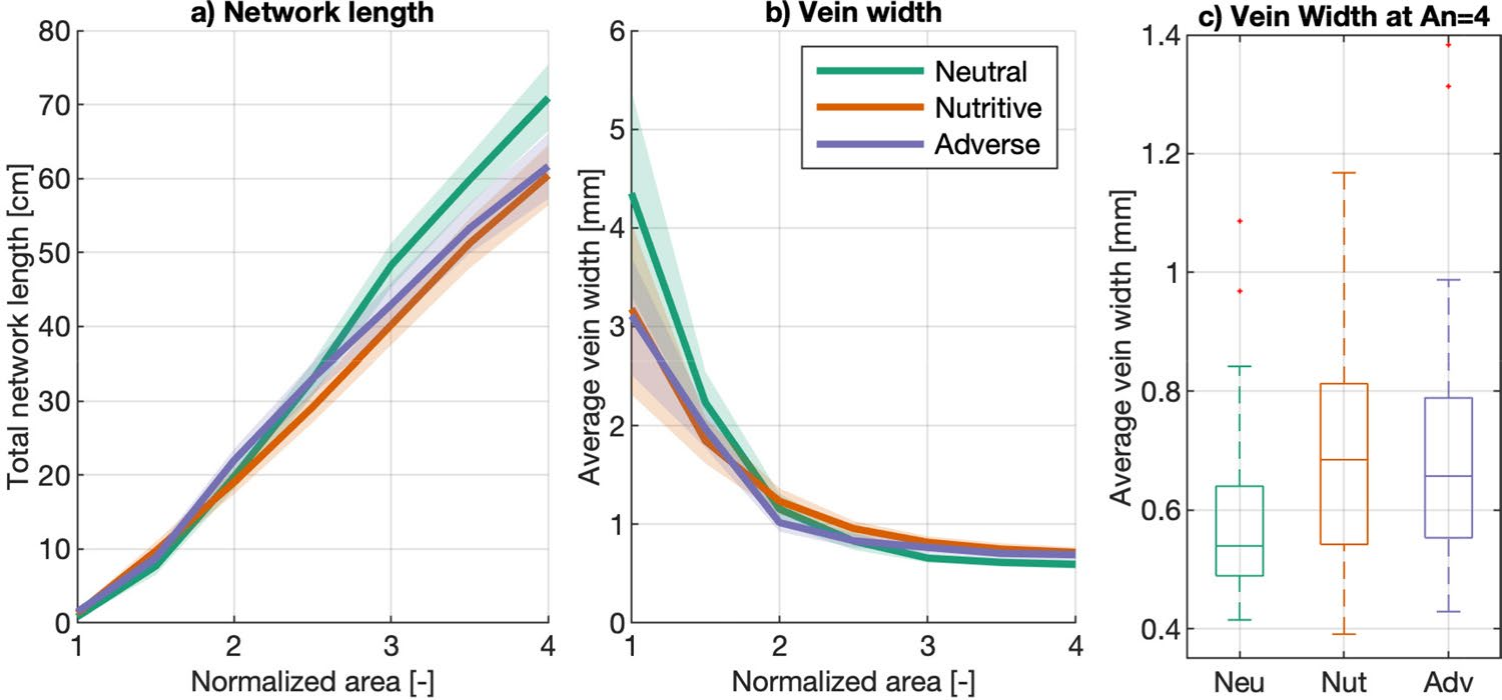
Total Network Length and average vein width. (**a**) total network length as a function of normalized slime mold cell area (An). (**b**) Average vein width of the network as a function of normalized slime mold cell area (An), computed as the ratio between the slime mold area and the total network length. (**c**) Boxplot graph of the average vein width at An = 4. The solid lines and shaded areas correspond to the means and confidence intervals among replicates, respectively. Box plots show median (horizontal line), interquartile range (box), distance from upper and lower quartiles times 1.5 interquartile range (whiskers), and outliers ($>1.5x$ upper or lower quartile).

Regarding the total length of the networks, slime molds migrating on a neutral substrate build longer networks (Table S7, Fig. 7a) with narrower veins (Tables S8–S9, Fig. 7b, right hand-side and Fig. 7c), more nodes (Table S10, Fig. 6) and more edges (Table S11, Fig. 6) than on the other two substrates.

We observe that when slime molds reach three times their initial area (An = 1), the vein width becomes constant. We also note a general decrease of vein width over the initial expansion from An = 1 to An = 3, which was expected, since the biomass remains constant in these environments^11^: pseudopods are gradually transformed into veins. The main interpretation is that on a neutral substrate, the print area (PA) of the slime molds is larger because the slime molds are exploring: there are more empty spaces, and there are more connections between nodes and between edges. Since we are comparing slime molds that have the same cell area (CA) (and density), veins have to be narrower than in the nutritive and adverse environments, by biomass conservation (Fig. 7).

**Figure 8:**
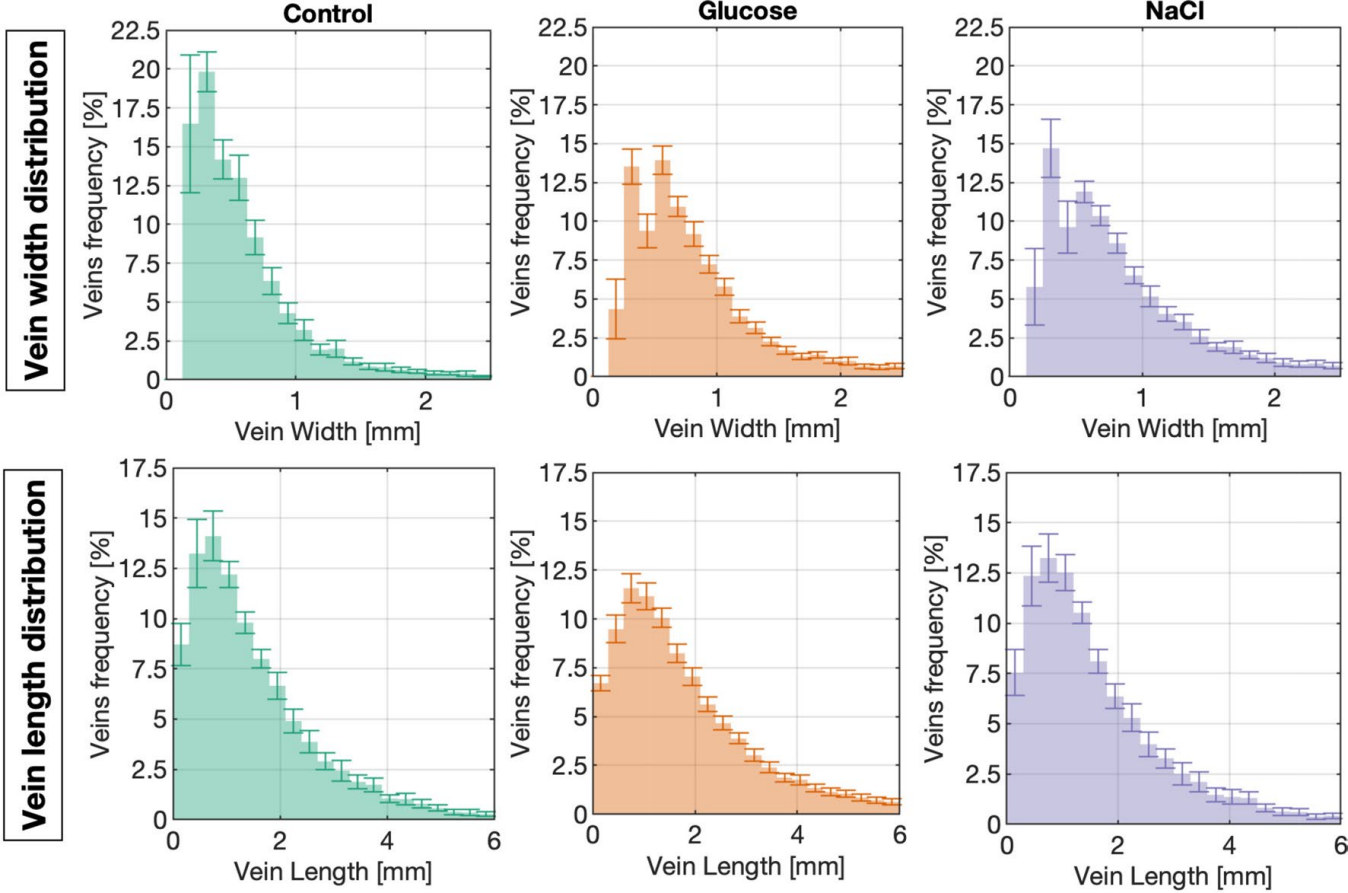
Distribution of veins width (top row) and length (bottom row). Bars in the frequency histograms show the mean percentage of network veins within a given range, while vertical error bars show the confidence interval.

The analysis of vein width and vein length distribution indicates that veins are the narrowest and the shortest when slime molds explore a neutral environment (Tables S12–S15, Fig. 8, Supplementary Figures S39–S40), following a log-logistic distribution with abundant short and thin veins and few long and thick veins.

**Figure 9:**
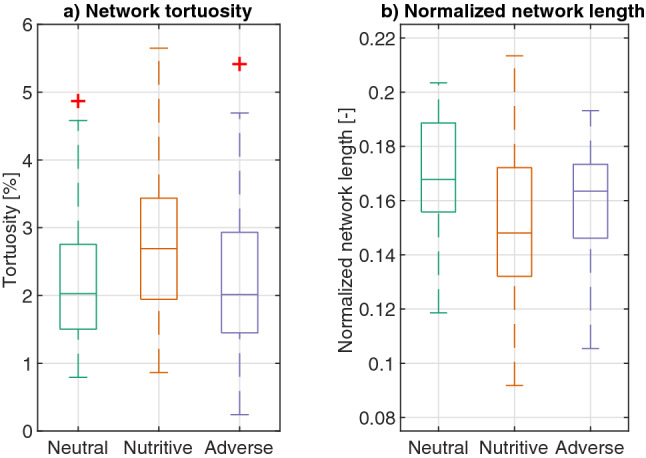
(**a**) Network tortuosity, computed by comparing the length of the network to the length of a network of equivalent topology but strictly straight segments between nodes. (**b**) Normalized network length, mapped to the interval [0 1], where the minimum spanning tree (MST) and Delaunay triangulation DT are the lower and upper bounds respectively. Box plots show median (horizontal line), interquartile range (box), distance from upper and lower quartiles times 1.5 interquartile range (whiskers), and outliers ($>1.5x$ upper or lower quartile).

The analysis of the normalized length of the networks shows that in general, slime mold networks have low tortuosity (e.g. edge lengths are close to the straight-line distances between nodes), with a slight difference in tortuosity and normalized network length between slime molds exploring a neutral substrate and slime molds exploring a nutritive one (Table S16, Table S17), the latter being the most tortuous (Fig. 9a), the former being the longest (Fig. 9b). Interestingly, we observe that the total length of the slime mold network is closer to that of a minimum spanning tree (which corresponds to a normalized length of zero) than to the total length of a fully connected network of the same topology (Delaunay triangulation, normalized length of 1), which at the same time, suggests that even though slime molds form networks with loops, those networks are far from being fully connected networks, keeping their total length closer to the MST.

**Figure 10:**
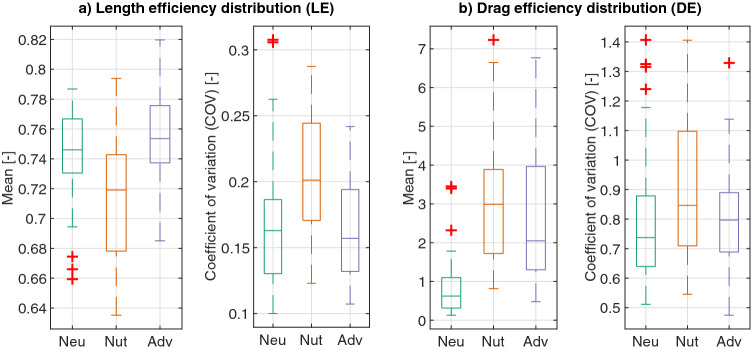
Transport efficiency. (**a**) Length efficiency distribution: ratio between the distance along the network and the Euclidean distance between every pair of nodes in the network. (**b**) Drag efficiency distribution: ratio between the drag along the network and along a straight path with a 0.5 mm width (arbitrary, constant value). The coefficient of variation (COV) corresponds to the standard deviation divided by the mean of the values. Box plots show median (horizontal line), interquartile range (box), distance from upper and lower quartiles times 1.5 interquartile range (whiskers), and outliers ($>1.5x$ upper or lower quartile).

The efficiency of the network measures how effective the network is to connect all its regions with one another. We calculate such efficiency in terms of path length (length efficiency, LE) and in terms of path drag (drag efficiency, DE). Drag can be understood as the resistance to flow between two points, and therefore a higher drag efficiency means higher ease of flow. The length efficiency is the lowest for the slime molds exploring a nutritive substrate (Table S18, Table S19, Fig. 10a) whereas the drag efficiency is the lowest for the slime molds exploring a neutral substrate (Table S20, Table S21, Fig. 10b).

**Figure 11:**
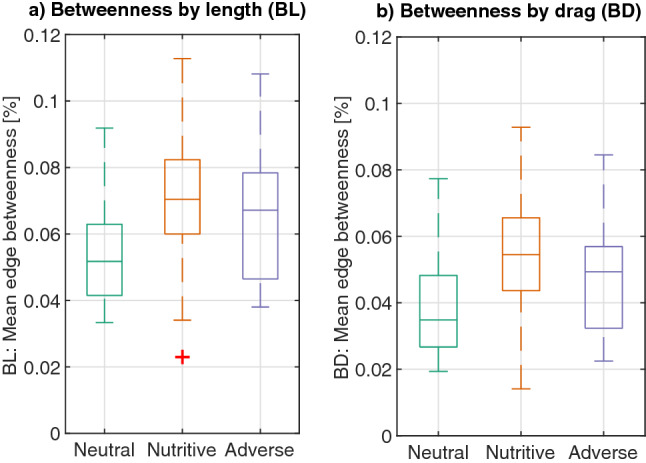
Edge betweenness: measure of edge importance, calculated as the percentage of shortest paths between nodes that pass through each edge. (**a**) Mean betweenness considering that the graph is weighted by the edge length (BL). (**b**) Mean betweenness considering that the graph is weighted by the edge drag (BD). Box plots show median (horizontal line), interquartile range (box), distance from upper and lower quartiles times 1.5 interquartile range (whiskers), and outliers ($>1.5x$ upper or lower quartile).

Another important metric of transportation networks is centrality, which is a measure of importance of the nodes or edges in the network, in terms of how often they are used during transport between locations. A homogeneous network has an even distribution of node/edge importance while a centralized network depends heavily on some important nodes/edges to route the network traffic. In the following, we measure edge importance in terms of betweenness centrality relative to both length and drag, which we calculate as the percentage of shortest paths (by length or drag) that pass through each node. The distribution of node importance in the networks (represented by mean edge betweenness), shows that slime molds in neutral environments are more homogeneous than those exploring nutritive and adverse environments. Slime molds in a nutritive environment are the most centralized among treatments (Tables S22–S23, Fig. 11a,b).

**Figure 12:**
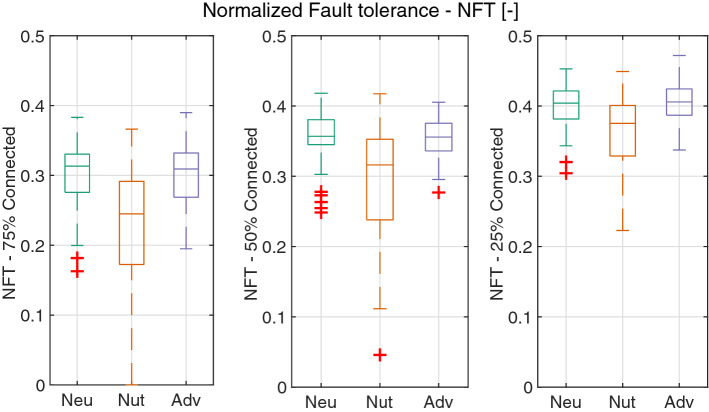
Normalized fault tolerances. The random fault tolerance (FT) is calculated as the percentage of edges that can be removed from a graph while still being able to connect $25\%$ (left), $50\%$ (middle) or $75\%$ (right) of the graph edges. The FT is normalized to the interval [0, 1] where the lower and upper bounds correspond to the minimum spanning tree (MST) and Delaunay triangulation (DT), respectively. The edges to remove are chosen randomly and therefore the average of 30 replicates is reported for each graph and its bounds. Box plots show median (horizontal line), interquartile range (box), distance from upper and lower quartiles times 1.5 interquartile range (whiskers), and outliers ($>1.5x$ upper or lower quartile).

Network resiliency, understood as the capacity of a network to perform adequately even with damage, is measured in terms of normalized fault tolerance. Fault tolerance is calculated as the percentage of randomly chosen edges that can be removed from the network while still connecting $25\%$, $50\%$ or $75\%$ of the nodes in the network. Fault tolerance is normalized to the interval [0, 1], where the bounds correspond to a simply connected (MST) and a maximally connected (DT) networks respectively. Results from Fig. 12 show that the networks in the neutral and adverse environments are similar in terms of resiliency, while the networks in the nutritive substrate are the least resilient (Tables S24, S25, S26, Fig. 12). The fact that networks in a nutritive environment are the most centralized and the least resilient suggest that they depend heavily on certain edges which sustain the connectivity of the network. On the contrary, the exploratory behaviour of slime molds in neutral or adverse conditions yields networks that are less centralized and more resilient, e.g. more capable to overcome accidental disconnections.

**Figure 13:**
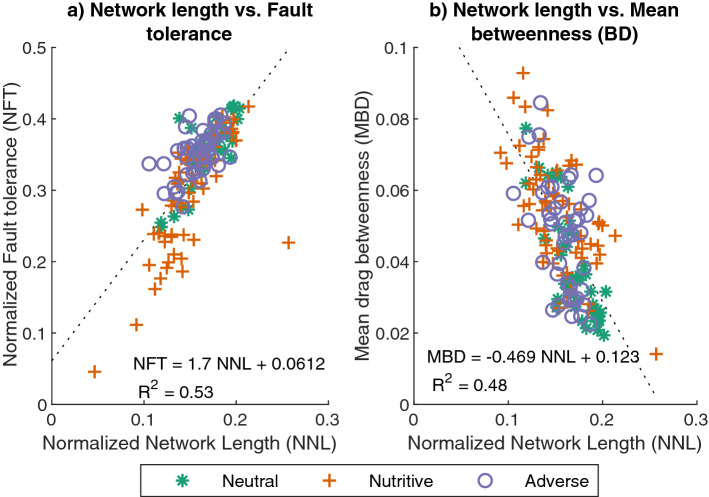
(**a**) Relationship between normalized network length (NNL) and random fault tolerance for $50\%$ connected nodes (FT50). (**b**) Relationship between normalized network length (NNL) and mean drag betweenness (BD).

Moreover, the relationships between network cost (normalized network length) and resiliency (normalized fault tolerance) and the edges importance distribution (mean drag betweenness) show that the normalized fault tolerance is positively correlated with the normalized network length (Table S27, Fig. 13a) whereas the mean drag betweenness is negatively correlated with the normalized network length (Table S28, Fig. 13b), Which shows that networks with higher network length have a more balanced importance distribution and are also more resilient.

**Figure 14:**
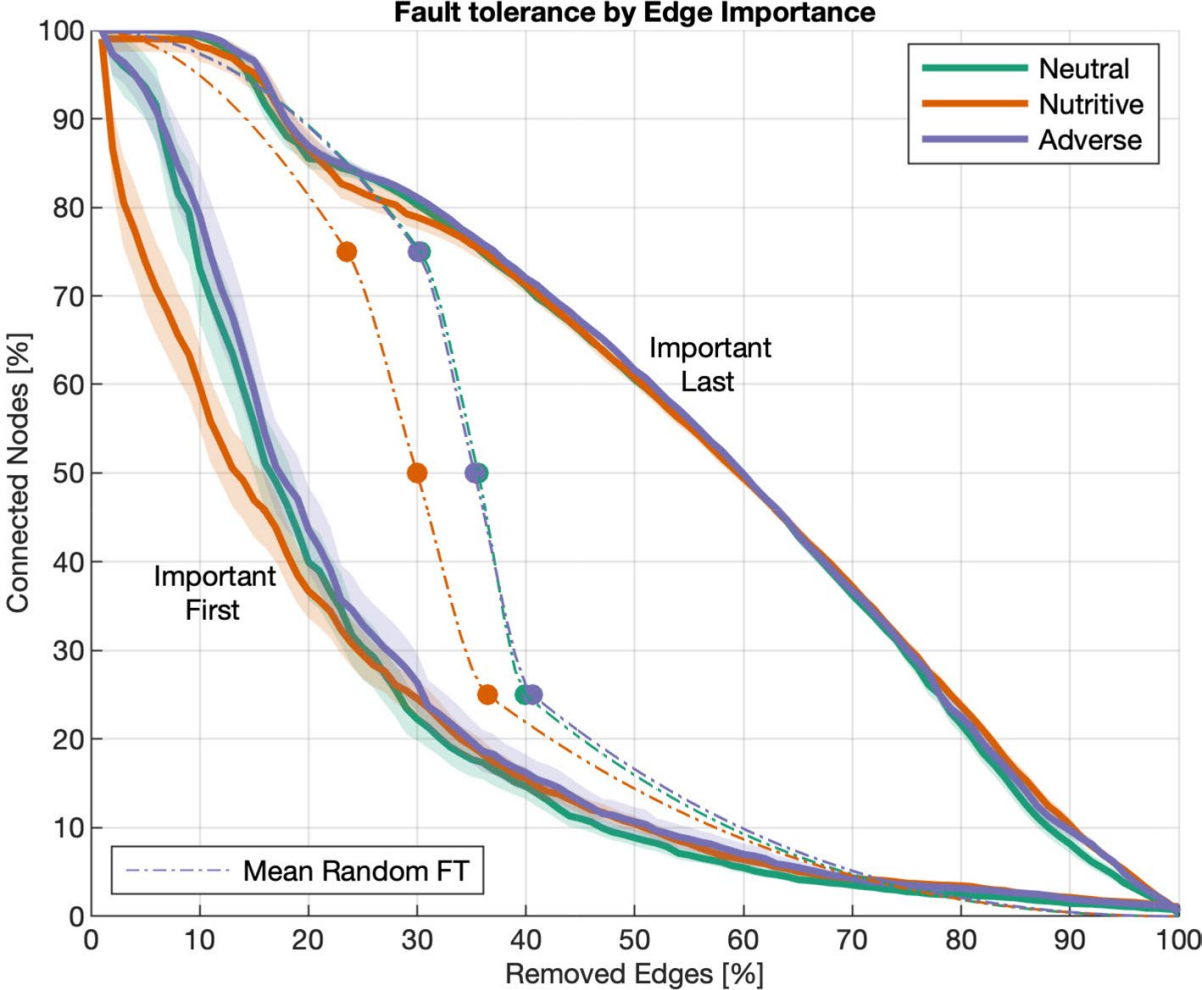
Fault tolerance Decay. Fault tolerance by edge importance. Decrease of the percentage of connected nodes in the graph (vertical axis) as a function of edge removal (horizontal axis). Dashed lines correspond to the mean random tolerance calculated for 25%, 50% and 75% of the nodes, connected by a spline. Solid lines and shaded regions correspond to the mean and confidence intervals (CI) for the worst and best case scenarios, resulting from removing the most important edges first and last, respectively.

Given that the normalized fault tolerance (NFT) is based on random removal of edges, it does not take into account the importance of the edges in the network (also called edge hierarchy). For this reason, it is interesting to calculate the decay of the number of nodes connected as a function of removed edges, where this time, the edges are sorted by their importance (drag betweenness) and then removed from the least (most) to the most (least) important edges, which correspond to the best (worse) case scenarios respectively for network resiliency. Results are shown in Fig. 14 together with the mean values of random fault tolerance shown in Fig. 12 (before normalization).

Results show that edge hierarchy significantly influences the fault tolerance of networks, showing a steep loss of connected nodes (around $70\%$ disconnected edges) after the $25\%$ of the most important edges are removed from the networks. This effect is even more considerable on the nutritive substrate, which is consistent with the fact that these networks are more centralized than those on the other substrates (see Fig. 11). However, regardless of the substrate, the percentage of connected edges remains almost unchanged (above $95\%$ of connected nodes) even after removing the $15\%$ of the edges with the lowest importance, which suggests that these edges are not essential to the flow of the network, but significantly contribute to resiliency. Regarding the random fault tolerance, the networks on neutral and adverse substrates show very similar resiliency while the networks on the nutritive substrate are more vulnerable to damage.

### Analysis of slime mold fusion process

**Figure 15:**
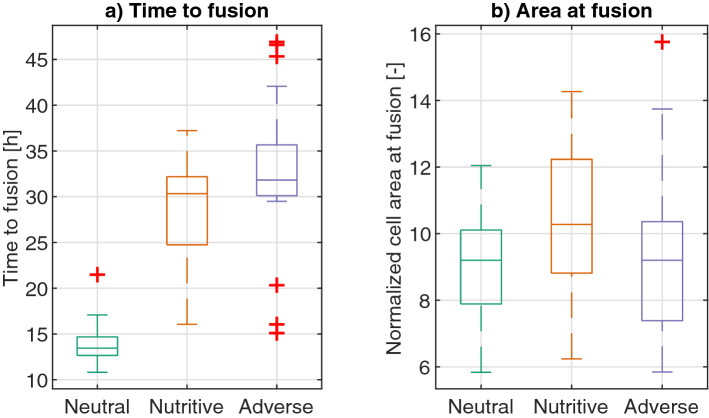
Time to fusion. (**a**) Boxplot with distribution of time elapsed from the beginning of the experiment until fusion between slime molds occurred. (**b**) Distribution of normalized areas (relative to initial cell area) of the slime mold cells at the time of fusion. Box plots show median (horizontal line), interquartile range (box), distance from upper and lower quartiles times 1.5 interquartile range (whiskers), and outliers ($>1.5x$ upper or lower quartile).

The second part of the analysis focuses on the fusion process between two slime mold cells growing on different substrates. We measured the time at which the slime molds fused and the area of the whole slime mold as soon as they fused. Networks on a neutral environment reach fusion faster, which is consistent with the expansion rates observed in Fig. 4. Nonetheless, the area of the slime mold cells at fusion exhibits no significant differences among treatments (Table S29), showing that the differences among treatments correspond to difference in expansion rates and not in slime mold cell morphology (Fig. 15).

**Figure 16:**
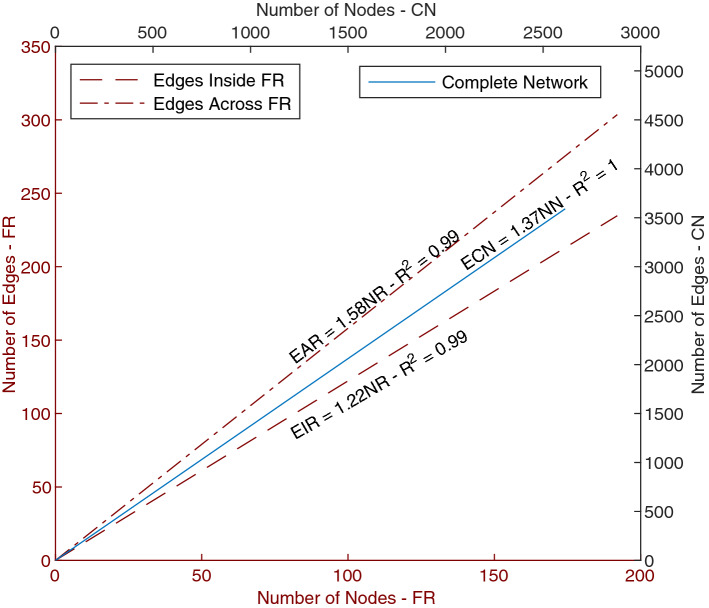
Number of nodes and edges inside the fusion region and the full network. Dashed lines (left and bottom axis) correspond to the relation between the number of nodes inside the fusion region (FR) and the number of edges inside it, e.g. both parent nodes of the edge lie inside FR, and number of edges across FR, i.e. at least one of the parent nodes of the edges lies inside FR. Solid line (right and top axis) correspond to the total number of nodes and edges in the complete, fused network.

In the following, we focus on the fusion region, i.e. the neighborhood where the slime mold cells fused at the first place. We tracked its characteristics for 3 h from the fusion time. First, we calculated the number of edges and nodes inside the fusion region. Figure 16 shows the relationship between nodes and edges within the whole network, and just inside the fusion region.

Results show that the relationship between nodes and edges in the whole network is similar to that observed in the individual slime mold cells (Fig. 6). Within the fusion region, this relationship depends on the way the edges inside the region are counted. But in any case, the node-to-edge relationship has a strong correlation irrespective of the substrate.

**Figure 17:**
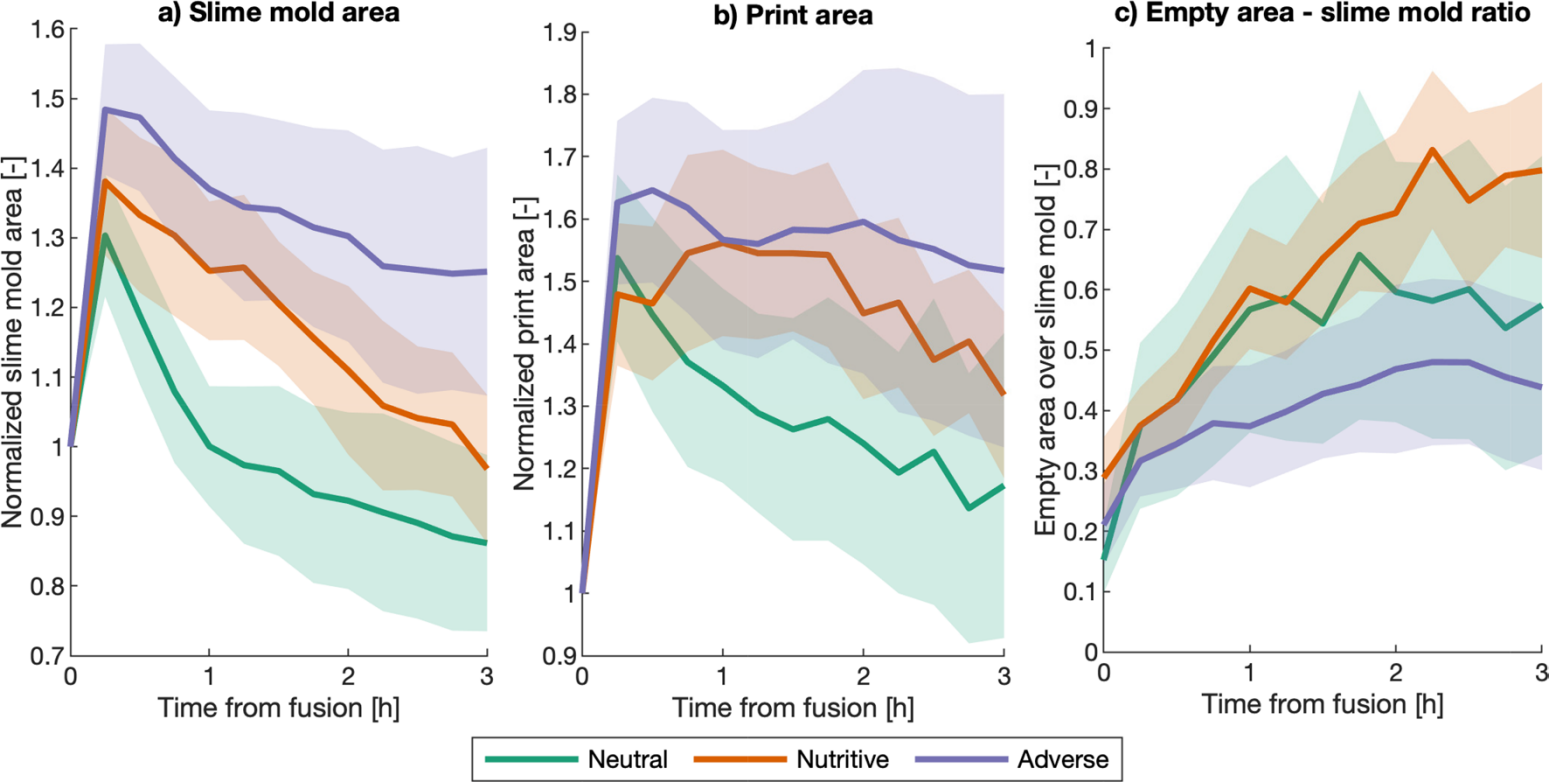
Area indexes inside the fusion region (FR). (**a**) slime mold area normalized by the area at fusion. (**b**) Print area of the slime mold cell (including enclosed empty space inside cell), normalized by the print area at fusion. (**c**) Ratio between enclosed empty space and slime mold areas.

Next, we study the morphology evolution of the slime mold cell within the fusion region. Figure 17 shows the change of slime mold area, print area and enclosed area.

Results have been normalized with respect to their values at fusion time. In general, the slime mold and print areas increase for a certain time after fusion, reaching a maximum, and then steadily decrease. Statistic analyses show that the rate at which the slime mold area decreases after fusion on a neutral substrate is significantly higher than the rate on the adverse and nutritive substrates (Table S30). Similarly, the rate at which the print area decreases in the neutral environment is higher than that in the nutritive environment (Table S30). Lastly, the ratio of empty area to slime mold area increases steadily for all the treatments, suggesting that the slime mold is refining, becoming less compact in the process. Slime mold is refining at a faster rate in the nutritive environment than in the neutral environment (Table S31).

**Figure 18:**
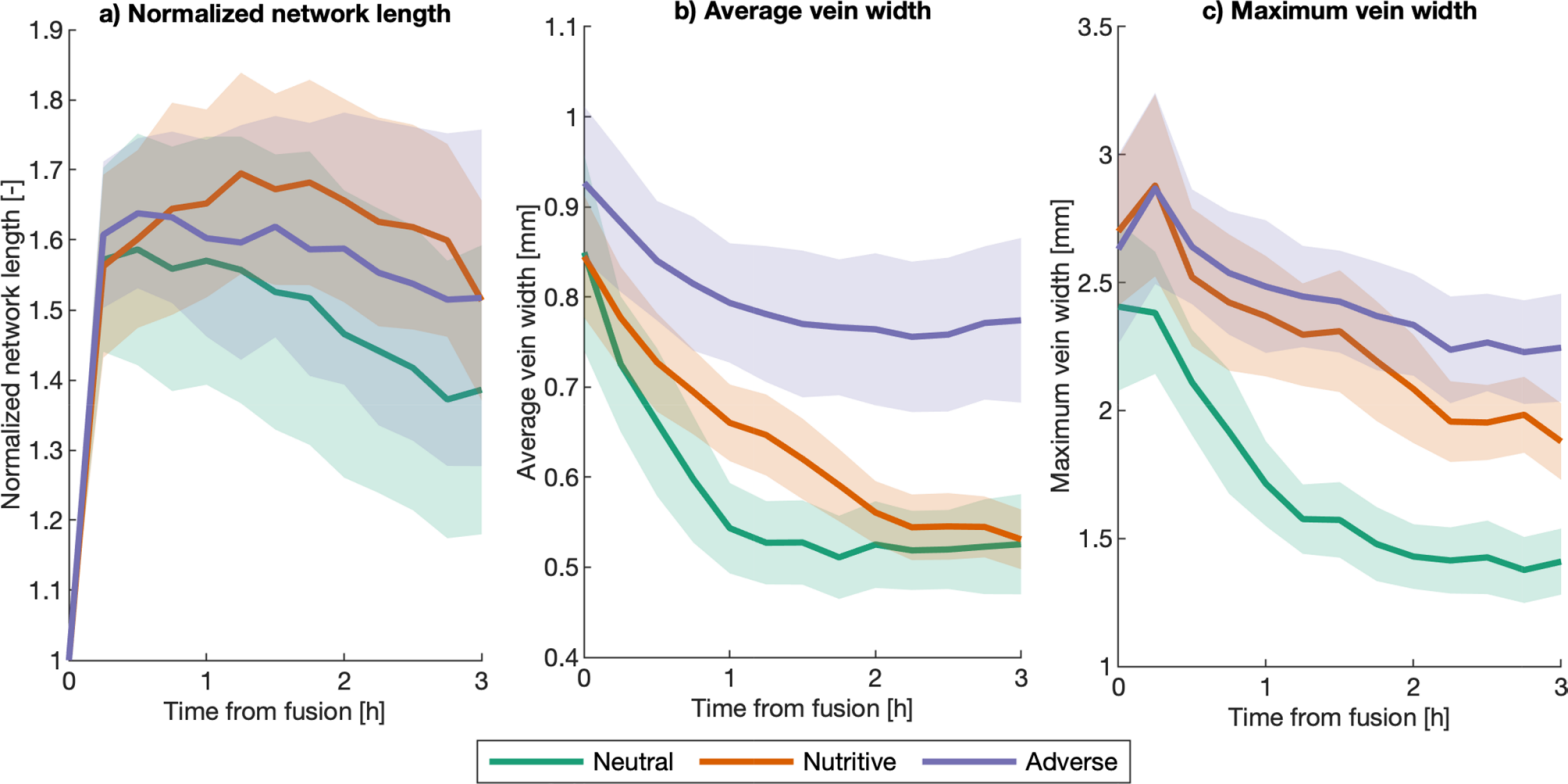
Network length and vein width inside FR (**a**) Network length inside normalized by the network length at fusion. (**b**) Average vein width inside FR. (**c**) Maximum vein width inside FR. Solid lines correspond to the mean value among replicates for the same substrate and shaded regions correspond to its confidence interval.

Results from Fig. 18a show that the network length significantly increases after fusion. Even though it eventually reduces, it is always higher than the initial network length at fusion. The refinement rate at which the network length decreases in the neutral environment is higher than that in the nutritive environment (Table S32). Refinement inside the fusion region is further observed through the evolution of the average and maximum vein widths (Fig. 18b,c), which reduce after fusion and eventually reach an equilibrium. Statistical analyses show that the rate at which the maximum and average vein widths decrease is faster in neutral environments compared to nutritive ones (Tables S33–S34). In addition, the maximum vein widths after 3 h from fusion are significantly lower on the neutral substrate and are significantly different in the nutritive environment compared to the other two (Table S34).

To analyze the evolution of the connectivity between the previously separated slime mold cells, we tracked the number and average width of the veins that connect both sides of the newly fused slime mold and we also tracked the path distance and the drag between both sides. The number and width of the connecting veins are found from the maximum flow of the graph, as described in the methods section.

**Figure 19:**
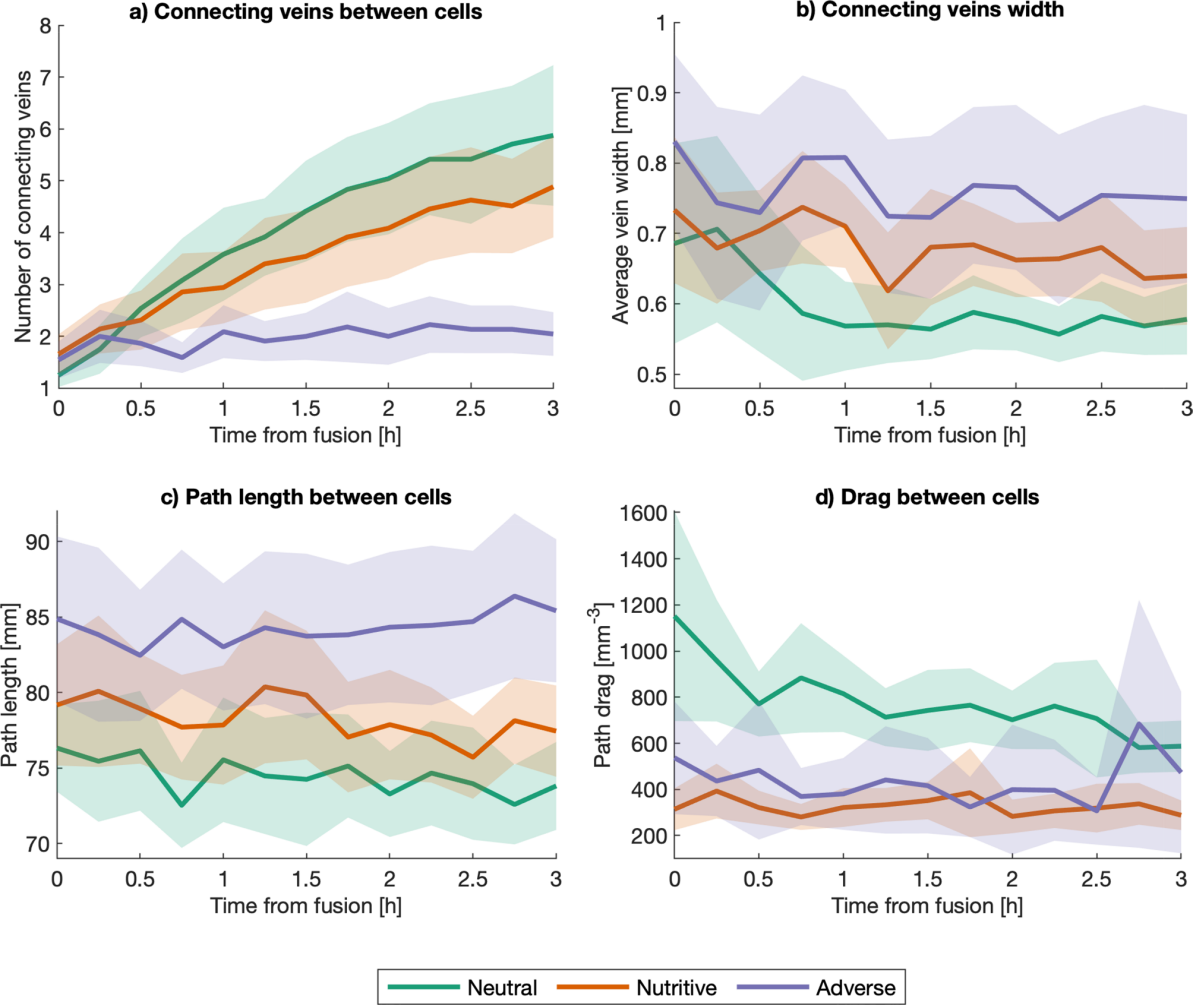
Connectivity between slime mold cells. (**a**) Number of connecting veins between fused slime mold cells. (**b**) Average width of the connecting veins, calculated as the total width of the region between slime mold cells, divided by the number of connecting veins. (**c**) Path length between initial slime mold cells: shortest distance between the two initial slime mold cells along the network. (**d**) Minimum drag along the path connecting the two initial slime mold cells in the network.

From Fig. 19a, we can see that the number of connecting veins in the neutral and nutritive environments consistently increases after fusion, while it remains relatively constant in the adverse environment, with between 1 and 2 veins connecting both sides of the initial slime mold cells (Table S35). The average width of the veins connecting the slime mold cells remains relatively constant after fusion (Fig. 19b), with the networks in the adverse environment showing a higher connecting vein thickness than the ones in the neutral environment (Table S36). These results suggest that slime molds in the neutral environment create several, relatively thin connecting veins between slime mold cells, in contrast to the adverse environment, in which the connection between slime mold cells depends on few (around 1 or 2) thicker veins.

Moreover, the shortest path between the connected slime mold cells is significantly longer in adverse environments, as shown in Fig. 19c (Table S37), which might be a consequence of the expansion dynamics of slime mold in such environments. A neutral environment fosters a ’spread-out’ network (Fig. 5), which is more likely to fuse with the other slime mold cell at mid-distance, eventually resulting in a shorter path between slime mold cells. On the contrary, the networks in adverse environments follow a more compact expansion direction, taking significantly more time to fuse (Fig. 15) and possibly resulting in fusing regions in more diverse locations, through which connecting veins develop, eventually resulting in longer connecting paths.

The drag between slime mold cells, shown in Fig. 19d, shows that even though the path length between slime mold cells in the neutral environment is lower than that in the adverse environment, the fact that the veins in the neutral environment are thinner (including the connecting veins) results in a higher drag between slime mold cells (Table S38). Moreover, while the drag and path lengths between slime mold cells remain relatively constant in the adverse environment, they decrease on the neutral substrate, suggesting the networks in the neutral environment further optimize the connection between slime mold cells after fusion (Tables S37–S38).

## Discussion

The analysis of slime mold networks and expansion dynamics with a semi-automated image processing approach confirms that substrates have a direct impact on the expansion rate of the cells^11,43^. Expansion slows down in nutritive and adverse environments (Fig. 4), which yields an earlier time to fusion in a neutral environment (Fig. 15). Based on a comparison of slime mold cells of same area, it was found that slime molds in the neutral conditions follow an exploratory behavior, creating spread-out networks quickly, while slime molds in adverse environments minimize their number of connections and expansion rate, to avoid exposure to the repellent substance and save energy^44^ , while still exploring the domain. On the contrary, slime molds in the nutritive environment seem to focus on metabolizing the glucose, pushing aside the exploratory behavior. This observation is supported by the total network length and average vein widths of the substrates (Fig. 7), which shows that slime molds in neutral environments build longer networks with thin veins, as they are more ’spread-out’, while nutritive and adverse substrates promote compact, denser networks. Hence, our findings confirm previous observations^22,26^ that network characteristics depend on the environmental conditions and our results are in agreement with previous observations on vein width^18,21^.

The connectivity of the different networks is not affected by the substrate or time period of expansion of the networks, and we observed a linear relationship between connectivity and the number of edges and nodes (number of edges around 1.35 times the number of edges), which supports the fact that the mean node degree of slime mold cells is a little below 3—a common feature of biological networks as previously shown by other authors^20,24^. Moreover, when comparing the networks to the theoretical bounds, e.g. simply (MST) and fully (DT) connected networks, we found that slime molds yield total lengths closer to simply connected networks with low tortuosity, suggesting a cost minimization strategy.

Slime mold networks contains loops, even though slime mold seems to minimize its network length, as demonstrated by a relatively high transport efficiency when compared to the straight distance between nodes. Such efficiency proved to be higher in neutral and adverse environments when compared to nutritive environments. Conversely, the drag efficiency of the networks built in the neutral substrate was the lowest, which is congruent with the fact that neutral environments promote a spread-out network (longer distances), with thinner veins (higher drag). The normalized network length (relative to the MST and DT) proved to be proportional to the normalized fault tolerance (resiliency) of the networks, while it was inversely proportional to the mean drag betweenness (network centrality). These findings show that longer networks (with the same total area) are more connected and therefore are more resilient and less centralized.

The similarity between the network dynamics of the networks built in neutral and adverse environments arises again when considering the load balance and resiliency of the networks. Slime molds in the nutritive environment produce more centralized networks, e.g. the traffic depends on few edges^24^, which also explains the fact that such networks are less resilient, becoming more disconnected as some edges disappear from the network^45^. This observation suggests that even though the slime molds in neutral and adverse environments have significantly different expansion rates, they still follow a similar exploration strategy, while the slime molds in the nutritive environment follow a different dynamic due to the presence of the glucose being absorbed.

By observing the fusion process between slime mold cells, we noted that after coming into contact with each other, usually via two pseudopods, a dense, fusion region develops. And after a certain point (about 20 min), that region starts thinning at different rates (faster in the neutral environment), going from a dense region to an array of connecting veins, thinner in the neutral environment. In terms of connectivity between fused cells, the fast exploration dynamics of the slime molds in a neutral environment results in a highly connected network, with several, thin connecting veins between the previously disconnected cells. On the contrary, the networks in the adverse environment remain connected with few (1 or 2 in average) but thick veins. Regarding the path length and drag between the connected cells in a neutral environment, the number of connecting veins between the two slime mold cells results in shorter (more efficient) paths, compared to the other two treatments. And even though veins in the neutral environment are generally thinner, their thickness evolves to reduce the drag between the connecting paths.

Through our study, we show that slime molds build elaborate networks that are highly responsive to environmental conditions. These networks develop as the organism explores its environment for new resources. In many vascular networks observed in living systems, efficiency corresponds to a measure of how fast nutrients and oxygen can be transported along the network, while cost measures how much energy or carbon is needed to construct such network. Slime mold networks, similarly to fungal networks, are built following local iterative expanding steps rather than a pre-planned blueprint^46^. The expansion involves building links and nodes in excess, followed by selective pruning and reinforcement of particular links, that refines the network to maximize the transport efficiency, modifying the hierarchy of the network edges^47^, and is consistent without centrality measurements (see Fig. 11). It has been demonstrated that coupling between strengthening and elimination in slime molds allows the network to transit from a fine mesh in a neutral environment to an optimal solution when food resources are added in the environment, as shown in previous studies^47,48^.

We observed that slime molds networks in exploration mode exhibit many loops. Loops are a common feature of natural networks such as as animal, plant and fungi vascular networks^49^. It has been suggested that the redundancy of vascular networks could be an adaptation to the varying physiological demands of different parts of the system and a way to withstand damages^50^. The existence of loops prevents disconnection of the network. In the absence of loop, severing a vein would result in the loss of all the network sections downstream from that vein. In contrast to plants and animals in which the vascular networks form only a small part of the organism and is usually protected and preserved from the environment, in both slime mold and fungi, the network defines the organism itself^46^. Hence, vascular networks in plants and animals are usually very efficient and short, but they require time to adapt to variable environmental conditions. In contrast, fungi and slime mold networks tend to be robust and flexible, but long and redundant^51^.

More broadly, deploying an efficient network in a constrained environment with a finite amount of resources is a common objective to both biological and human-made systems. Regardless of scale, ranging from ecosystems and communities, such as ant colonies, to simple organisms and organs, such as neural arbors and vascular systems, it has been observed that network deployment strategies seek to maximize travel efficiency and/or redundancy (and therefore resiliency) while minimizing cost. Commonalities between natural and engineered networks have originated the field of bio-inspiration, which aims to mimic biological principles to improve the design and construction of human-made systems. For instance, the principles of networks scale have shown to be applicable to both biological, human, and geographic networks expanding the similarities between networks to the principles behind the formation of basins and hydrology systems^52^. Comparison between biological and computational networks showed the differences between sparse and robust networks in terms of connectivity and optimization strategies^53^ and highlighted the potential to combine biological principles and engineering control to yield more efficient networks than those created with each strategy alone^54^. Other studies have focused on transportation networks. For instance, Patino-Ramirez et al.^55^ showed that a bio-inspired road network design based on leaf venations can reduce the unitary construction cost of the networks, while yielding similar path efficiencies to those obtained with global optimum networks, such as Steiner Trees. Similarly, Tero et al.^40^, showed that slime mold creates efficient algorithms in terms of cost, path efficiency and resiliency, that can outperform current design methods used to design railway systems.

Civil infrastructure networks are traditionally designed to optimize objective functions (such as traffic flow) under static constraints (such as land use regulations). Network resilience and security have recently gained attention in several fields of engineering that study the impact of global threats such as pandemics, terrorism or climate change^56^. It has become increasingly important to design networks that adapt to disruptions (e.g., cut connection after a natural disaster) or attacks (e.g., data hacking or spread of a disease). In this context, classical optimization under constraint is not applicable. Slime mold networks extend beyond the boundaries of a given set of nodes, and continue to extend during optimization of the vein network left behind. Slime mold is a good model organism to study network optimization under perpetual expansion. A possible application is the necessary adaptation of networks of utilities as urban areas grow or recess or the redistribution of flow in networks used cyclically or seasonally. For example, the direction of road lanes is inverted twice a day in some large cities like Washington DC. Storm water management relies on the simulation of weather scenarios, in which the final design option, e.g. pipe upsizing, underground storage, or bio-filtration, remains empirical^57,58^. Probabilistic approaches were used to assess the vulnerability of power systems^59^ and virtual networks^60^, such as internet and optical networks, to physical infrastructure damage. The most recent publications treat the resilience of inter-related networks such as power, water and cellular networks^61^. In all of these models, analyses are done *a posteriori*. More dynamic approaches based on the game theory were adopted to secure infrastructure and information networks, whereby the cost of creating and removing links in a graph is calculated at every move of the attacker or the defender^62^. No strategy has been proposed yet to account for the splitting and fusion functions of slime mold networks, which relate to specialization versus generalization behavior. How to split water networks to ensure autonomy of communities in remote areas? When should two countries merge electrical power grids after a hurricane? So far, these questions are open. To the authors’ best knowledge, the only studies available to date focus on data fusion^63–65^ or on emergency operation center fusion^66^. The dynamics of slime mold networks before and after fusion in response to various environmental constraints has a potential to inspire new strategies to design adaptable information and infrastructure networks, resilient to natural and biological hazards as well as geopolitical risks.

## Concluding statement

Slime molds are a good model organism to study networks. First, slime mold networks are both a transport system and the organism itself, and are therefore more dynamic than vascular networks in plants and animals. Second, slime mold networks adopt an array of topologies in response to environmental conditions. Third, slime mold networks can be continuously remodelled via pruning and reinforcement, adapting to different nutrient conditions and damage. Fourth, slime mold networks differ from other types of vascular networks, due to an unlimited capacity for expansion, combined with an ability to maintain its functionality as a living unit. Lastly, self-fusion yields optimised interconnected networks.

## Supplementary information

Supplementary information 1

Supplementary information 2

Supplementary information 3

Supplementary information 4

Supplementary information 5

Supplementary information 6

Supplementary information 7

## Data Availability

The index datasets generated and analysed during the current study are included in this published article as Supplementary Information files. Raw images data is available from the corresponding author on reasonable request.
