# Supplementary material for: Substrate and cell fusion influence on slime mold network dynamics
Source: Sci Rep. 2021 Jan 15;11:1498. doi: 10.1038/s41598-020-80320-2 (PMC7810851; doi:10.1038/s41598-020-80320-2)
Supplement: Supplementary file 4 — Supplementary information 4 [file 41598_2020_80320_MOESM4_ESM.pdf]

Supplementary Information: Substrate and cell fusion influence on Slime mold network dynamics

Fernando Patino-Ramirez, Chloé Arson, Audrey Dussutour

Statistical analysis

	Response Variable: Time elapsed to normalized area				
Predictors (Explanatory variables)	*Estimates*	*CI*	*Statistic*	*p*	*df*
Intercept	-1.90	-2.09 – -1.72	-20.33	**<0.001**	1351.00
[Neutral] vs [Nutritive]	-0.09	-0.26 – 0.08	-1.05	0.292	1351.00
[Neutral] vs [Adverse]	-0.55	-0.72 – -0.38	-6.35	**<0.001**	1351.00
Normalized area [An]	0.58	0.54 – 0.63	25.62	**<0.001**	1351.00
[An]:[Neutral] vs [An]:[Nutritive]	0.24	0.19 – 0.30	8.29	**<0.001**	1351.00
[An]:[Neutral] vs [An]:[Adverse]	0.47	0.41 – 0.53	15.33	**<0.001**	1351.00
**Random Effects**					
Within-group variance σ^2	0.18				
Between-group-variance $\tau_{00\ ID:Plasmodium}$	0.04				
Between-group-variance $\tau_{00\ Plasmodium}$	0.05				
Intraclass-Correlation Coefficient	0.34				
N_{ID}	206				
$N_{Plasmodium}$	12				
Observations	1360				
Marginal R^2 / Conditional R^2	0.734 / 0.824				

Table S1: Time elapsed to normalized area. Statistics associated to Figure 4.
To assess the difference in time elapsed to normalized area between the three treatments; we used a linear mixed model (function lmer, Package lme4). The model was fitted by specifying the fixed effects: treatment (categorical predictor) and normalized area (continuous predictor) and the random effects: Plasmodium and Replicate. The dependent variable (Time) was normalized using the function bestNormalize (Package bestNormalize). N> 50 replicates for each treatment.
An = Normalized Area; N_{ID} = Slime mold Identity, $N_{Plasmodium}$ Plasmodium Identity

	Response Variable: Empty space over slime mold area				
Predictors (Explanatory variables)	*Estimates*	*CI*	*Statistic*	*p*	*df*
Intercept	-2.34	-2.48 – -2.19	-31.30	**<0.001**	1351.00
[Neutral] vs [Nutritive]	0.18	0.02 – 0.34	2.25	**0.024**	1351.00
[Neutral] vs [Adverse]	0.20	0.04 – 0.37	2.43	**0.015**	1351.00
Normalized area [An]	1.05	1.00 – 1.09	45.57	**<0.001**	1351.00
[An]:[Neutral] vs [An]:[Nutritive]	-0.19	-0.25 – -0.13	-6.40	**<0.001**	1351.00
[An]:[Neutral] vs [An]:[Adverse]	-0.21	-0.27 – -0.15	-6.70	**<0.001**	1351.00
**Random Effects**					
Within-group variance σ^2	0.18				
Between-group-variance $\tau_{00\ ID:Plasmodium}$	0.02				
Between-group-variance $\tau_{00\ Plasmodium}$	0.02				
Intraclass-Correlation Coefficient	0.16				
N_{ID}	206				
$N_{Plasmodium}$	12				
Observations	1360				
Marginal R^2 / Conditional R^2	0.786 / 0.820				

Table S2: Empty space over slime mold area. Statistics associated to Figure 5a.
To assess the difference in empty space over slime mold area between the three treatments; we used a linear mixed model (function lmer, Package lme4). The model was fitted by specifying the fixed effects: treatment (categorical predictor) and normalized area (continuous predictor) and the random effects: Plasmodium and Replicate. The dependent variable (empty space over slime mold area) was normalized using the function bestNormalize (Package

bestNormalize). N> 50 replicates for each treatment. An = Normalized Area; N_{ID} = Slime mold Identity, $N_{Plasmodium}$ Plasmodium Identity.

	**Response Variable: Number of empty regions**				
Predictors (Explanatory variables)	*Incidence Rate Ratios*	*CI*	*Statistic*	*p*	*df*
Intercept	191.96	147.34 – 250.11	38.94	**<0.001**	149.00
[Neutral] vs [Nutritive]	0.65	0.63 – 0.68	-23.87	**<0.001**	149.00
[Neutral] vs [Adverse]	0.90	0.87 – 0.93	-5.85	**<0.001**	149.00
**Random Effects**					
Within-group variance σ^2	0.01				
Between-group-variance $\tau_{00\ Plasmodium}$	0.22				
Intraclass-Correlation Coefficient	0.97				
$N_{Plasmodium}$	12				
Observations	153				
Marginal R^2 / Conditional R^2	0.139 / 0.976				

Table S3: Number of empty regions. Statistics associated to Figure 5b.
To assess the difference in the number of empty regions between the three treatments; we used a generalized linear mixed model (function glmer, Package lme4). The model was fitted by specifying the fixed effects: treatment (categorical predictor) and the random effect: Plasmodium. N> 50 replicates for each treatment.
$N_{Plasmodium}$ Plasmodium Identity.

	**Response Variable: Mean area of the empty regions**				
Predictors (Explanatory variables)	*Estimates*	*CI*	*Statistic*	*p*	*df*
Intercept	-0.30	-0.76 – 0.17	-1.26	0.208	148.00
[Neutral] vs [Nutritive]	-0.11	-0.38 – 0.15	-0.85	0.393	148.00
[Neutral] vs [Adverse]	-0.52	-0.81 – -0.24	-3.57	**<0.001**	148.00
**Random Effects**					
Within-group variance σ^2	0.42				
Between-group-variance $\tau_{00\ Plasmodium}$	0.54				
Intraclass-Correlation Coefficient	0.56				
$N_{Plasmodium}$	12				
Observations	153				
Marginal R^2 / Conditional R^2	0.044 / 0.580				

Table S4: Mean area of the empty regions when the normalized area is 4. Statistics associated to Figure 5c.
To assess the difference in empty space area between the three treatments; we used a linear mixed model (function lmer, Package lme4). The model was fitted by specifying the fixed effects: treatment (categorical predictor) and the random effect: Plasmodium. The dependent variable (empty space area) was normalized using the function bestNormalize (Package bestNormalize). N> 50 replicates for each treatment. $N_{Plasmodium}$ Plasmodium Identity.

	Response Variable: Proportion of pseudopods				
Predictors (Explanatory variables)	*Estimates*	*CI*	*Statistic*	*p*	*df*
Intercept	2.35	2.19 – 2.51	28.27	**<0.001**	1351.00
[Neutral] vs [Nutritive]	-0.37	-0.56 – -0.18	-3.80	**<0.001**	1351.00
[Neutral] vs [Adverse]	-0.22	-0.42 – -0.03	-2.21	**0.027**	1351.00
Normalized area [An]	-0.98	-1.03 – -0.94	-40.37	**<0.001**	1351.00
[An]:[Neutral] vs [An]:[Nutritive]	0.22	0.16 – 0.28	7.00	**<0.001**	1351.00
[An]:[Neutral] vs [An]:[Adverse]	0.10	0.04 – 0.17	3.08	**0.002**	1351.00
**Random Effects**					
σ^2	0.20				
$\tau_{00\ ID:Plasmodium}$	0.08				
$\tau_{00\ Plasmodium}$	0.01				
ICC	0.32				
N_{ID}	206				
$N_{Plasmodium}$	12				
Observations	1360				
Marginal R^2 / Conditional R^2	0.704 / 0.799				

Table S5: Proportion of pseudopods
To assess the difference in the proportion of pseudopods between the three treatments; we used a linear mixed model (function lmer, Package lme4). The model was fitted by specifying the fixed effects: treatment (categorical predictor) and normalized area (continuous predictor) and the random effects: Plasmodium and Replicate. The dependent variable (proportion of pseudopods) was normalized using the function bestNormalize (Package bestNormalize). N> 50 replicates for each treatment. An = Normalized Area; N_{ID} = Slime mold Identity, $N_{Plasmodium}$ Plasmodium Identity.

	Response Variable: Proportion of pseudopods when the normalized area is 4				
Predictors (Explanatory variables)	*Estimates*	*CI*	*Statistic*	*p*	*df*
Intercept	-0.31	-0.62 – 0.01	-1.92	0.054	148.00
[Neutral] vs [Nutritive]	0.59	0.21 – 0.96	3.08	**0.002**	148.00
[Neutral] vs [Adverse]	0.32	-0.09 – 0.73	1.53	0.127	148.00
**Random Effects**					
σ^2	0.91				
$\tau_{00\ Plasmodium}$	0.05				
ICC	0.05				
$N_{Plasmodium}$	12				
Observations	153				
Marginal R^2 / Conditional R^2	0.058 / 0.106				

Table S6: Proportion of pseudopods when the normalized area is 4
To assess the difference in the proportion of pseudopods between the three treatments; we used a linear mixed model (function lmer, Package lme4). The model was fitted by specifying the fixed effects: treatment (categorical predictor) and the random effect: Plasmodium. The dependent variable (proportion of pseudopods) was normalized using the function bestNormalize (Package bestNormalize). N> 50 replicates for each treatment. $N_{Plasmodium}$ Plasmodium Identity.

	Response Variable: Network length				
Predictors (Explanatory variables)	*Estimates*	*CI*	*Statistic*	*p*	*df*
Intercept	-2.47	-2.58 – -2.37	-45.57	**<0.001**	1351.00
[Neutral] vs [Nutritive]	0.25	0.11 – 0.39	3.56	**<0.001**	1351.00
[Neutral] vs [Adverse]	0.22	0.08 – 0.36	3.07	**0.002**	1351.00
Normalized area [An]	1.04	1.01 – 1.08	62.32	**<0.001**	1351.00
[An]:[Neutral] vs [An]:[Nutritive]	-0.15	-0.19 – -0.11	-6.87	**<0.001**	1351.00
[An]:[Neutral] vs [An]:[Adverse]	-0.10	-0.14 – -0.06	-4.41	**<0.001**	1351.00
**Random Effects**					
Within-group variance σ^2	0.10				
Between-group-variance $\tau_{00\ ID:Plasmodium}$	0.05				
Between-group-variance $\tau_{00\ Plasmodium}$	0.00				
N_{ID}	206				
$N_{Plasmodium}$	12				
Observations	1360				
Marginal R^2 / Conditional R^2	0.899 / NA				

Table S7: Total Network length. Statistics associated to Figure 7a.
To assess the difference in network length between the three treatments; we used a linear mixed model (function lmer, Package lme4). The model was fitted by specifying the fixed effects: treatment (categorical predictor) and normalized area (continuous predictor) and the random effects: Plasmodium and Replicate. The dependent variable (total network length) was normalized using the function bestNormalize (Package bestNormalize). N> 50 replicates for each treatment. An = Normalized Area; N_{ID} = Slime mold Identity, $N_{Plasmodium}$ Plasmodium Identity.

	Response Variable: Mean vein width				
Predictors (Explanatory variables)	*Estimates*	*CI*	*Statistic*	*p*	*df*
Intercept	2.50	2.28 – 2.72	22.43	**<0.001**	1199.00
[Neutral] vs [Nutritive]	-0.56	-0.85 – -0.28	-3.89	**<0.001**	1199.00
[Neutral] vs [Adverse]	-0.46	-0.75 – -0.17	-3.12	**0.002**	1199.00
Normalized area [An]	-1.01	-1.07 – -0.94	-30.62	**<0.001**	1199.00
[An]:[Neutral] vs [An]:[Nutritive]	0.30	0.22 – 0.38	7.08	**<0.001**	1199.00
[An]:[Neutral] vs [An]:[Adverse]	0.20	0.11 – 0.29	4.52	**<0.001**	1199.00
**Random Effects**					
Within-group variance σ^2	0.26				
Between-group-variance $\tau_{00\ ID:Plasmodium}$	0.22				
Between-group-variance $\tau_{00\ Plasmodium}$	0.00				
N_{ID}	206				
$N_{Plasmodium}$	12				
Observations	1208				
Marginal R^2 / Conditional R^2	0.676 / NA				

Table S8: Average vein width. Statistics associated to Figure 7b.
To assess the difference in mean vein width between the three treatments; we used a linear mixed model (function lmer, Package lme4). The model was fitted by specifying the fixed effects: treatment (categorical predictor) and normalized area (continuous predictor) and the random effects: Plasmodium and Replicate. The dependent variable (mean vein width) was normalized using the function bestNormalize (Package bestNormalize). N> 50 replicates for each treatment. An = Normalized Area; N_{ID} = Slime mold Identity, $N_{Plasmodium}$ Plasmodium Identity.

	Response Variable: Mean vein width (An = 4)				
Predictors (Explanatory variables)	*Estimates*	*CI*	*Statistic*	*p*	*df*
Intercept	-0.50	-0.83 – -0.18	-3.01	**0.003**	148.00
[Neutral] vs [Nutritive]	0.57	0.20 – 0.94	3.02	**0.002**	148.00
[Neutral] vs [Adverse]	0.53	0.13 – 0.93	2.59	**0.010**	148.00
**Random Effects**					
Within-group variance σ^2	0.87				
Between-group-variance $\tau_{00\ Plasmodium}$	0.09				
Intraclass-Correlation Coefficient	0.09				
$N_{Plasmodium}$	12				
Observations	153				
Marginal R^2 / Conditional R^2	0.062 / 0.147				

Table S9: Average vein width for AN=4. Statistics associated to Figure 7c.
To assess the difference in mean vein width between the three treatments; we used a linear mixed model (function lmer, Package lme4). The model was fitted by specifying the fixed effects: treatment (categorical predictor) and the random effect: Plasmodium. The dependent variable (mean vein width) was normalized using the function bestNormalize (Package bestNormalize). N> 50 replicates for each treatment. $N_{Plasmodium}$ Plasmodium Identity.

	Response Variable: Number of nodes				
Predictors (Explanatory variables)	*Incidence Rate Ratios*	*CI*	*Statistic*	*p*	*df*
Intercept	437.80	343.75 – 557.57	49.29	**<0.001**	149.00
[Neutral] vs [Nutritive]	0.72	0.70 – 0.73	-28.74	**<0.001**	149.00
[Neutral] vs [Adverse]	0.92	0.90 – 0.94	-7.35	**<0.001**	149.00
**Random Effects**					
Within-group variance σ^2	0.00				
Between-group-variance $\tau_{00\ Plasmodium}$	0.18				
Intraclass-Correlation Coefficient	0.99				
$N_{Plasmodium}$	12				
Observations	153				
Marginal R^2 / Conditional R^2	0.105 / 0.987				

Table S10: Number of nodes. Statistics associated to Figure 6.
To assess the difference in the number of nodes between the three treatments; we used a generalized linear mixed model (function glmer, Package lme4). The model was fitted by specifying the fixed effects: treatment (categorical predictor) and the random effect: Plasmodium. N> 50 replicates for each treatment. $N_{Plasmodium}$ Plasmodium Identity.

	Response Variable: Number of edges				
Predictors (Explanatory variables)	*Incidence Rate Ratios*	*CI*	*Statistic*	*p*	*df*
Intercept	592.12	461.56 – 759.60	50.23	**<0.001**	149.00
[Neutral] vs [Nutritive]	0.70	0.69 – 0.71	-35.46	**<0.001**	149.00
[Neutral] vs [Adverse]	0.91	0.89 – 0.93	-9.07	**<0.001**	149.00
**Random Effects**					
Within-group variance σ^2	0.00				
Between-group-variance $\tau_{00\ Plasmodium}$	0.19				
Intraclass-Correlation Coefficient	0.99				
$N_{Plasmodium}$	12				
Observations	153				
Marginal R^2 / Conditional R^2	0.112 / 0.991				

Table S11: Number of edges. Statistics associated to Figure 6.
To assess the difference in the number of edges between the three treatments; we used a generalized linear mixed model (function glmer, Package lme4). The model was fitted by specifying the fixed effects: treatment (categorical predictor) and the random effect: Plasmodium. N> 50 replicates for each treatment. $N_{Plasmodium}$ Plasmodium Identity.

	Response Variable: miu parameter, vein width				
Predictors (Explanatory variables)	*Estimates*	*CI*	*Statistic*	*p*	*df*
Intercept	-1.10	-1.47 – -0.72	-5.74	**<0.001**	148.00
[Neutral] vs [Nutritive]	0.98	0.75 – 1.22	8.20	**<0.001**	148.00
[Neutral] vs [Adverse]	1.11	0.86 – 1.37	8.55	**<0.001**	148.00
**Random Effects**					
Within-group variance σ^2	0.34				
Between-group-variance $\tau_{00\ Plasmodium}$	0.34				
Intraclass-Correlation Coefficient	0.50				
$N_{Plasmodium}$	12				
Observations	153				
Marginal R^2 / Conditional R^2	0.247 / 0.624				

Table S12: miu parameter, vein width. Statistics associated to Figure 8.
To assess the difference in miu parameter extracted from the log-logistic fit of the vein width distribution between the three treatments; we used a generalized linear mixed model (function glmer, Package lme4). The model was fitted by specifying the fixed effects: treatment (categorical predictor) and the random effect: Plasmodium. N> 50 replicates for each treatment. $N_{Plasmodium}$ Plasmodium Identity.

	Response Variable: Sigma parameter, vein width				
Predictors (Explanatory variables)	*Estimates*	*CI*	*Statistic*	*p*	*df*
Intercept	-0.07	-0.47 – 0.34	-0.33	0.745	148.00
[Neutral] vs [Nutritive]	0.54	0.20 – 0.88	3.14	**0.002**	148.00
[Neutral] vs [Adverse]	0.58	0.21 – 0.95	3.09	**0.002**	148.00
**Random Effects**					
Within-group variance σ^2	0.70				
Between-group-variance $\tau_{00\ Plasmodium}$	0.30				
Intraclass-Correlation Coefficient	0.30				
$N_{Plasmodium}$	12				
Observations	153				
Marginal R^2 / Conditional R^2	0.059 / 0.339				

Table S13: sigma parameter, vein width. Statistics associated to Figure 8.
To assess the difference in sigma parameter extracted from the log-logistic fit of the vein width distribution between the three treatments; we used a generalized linear mixed model (function glmer, Package lme4). The model was fitted by specifying the fixed effects: treatment (categorical predictor) and the random effect: Plasmodium. N> 50 replicates for each treatment. $N_{Plasmodium}$ Plasmodium Identity.

	Response Variable: miu parameter, vein length				
Predictors (Explanatory variables)	*Estimates*	*CI*	*Statistic*	*p*	*df*
Intercept	-0.58	-1.04 – -0.12	-2.49	**0.013**	148.00
[Neutral] vs [Nutritive]	0.34	0.09 – 0.60	2.66	**0.008**	148.00
[Neutral] vs [Adverse]	-0.05	-0.32 – 0.23	-0.33	0.741	148.00
**Random Effects**					
Within-group variance σ^2	0.39				
Between-group-variance $\tau_{00\ Plasmodium}$	0.54				
Intraclass-Correlation Coefficient	0.58				
$N_{Plasmodium}$	12				
Observations	153				
Marginal R^2 / Conditional R^2	0.035 / 0.598				

Table S14: miu parameter, vein length.
To assess the difference in miu parameter extracted from the log-logistic fit of the vein length distribution between the three treatments; we used a generalized linear mixed model (function glmer, Package lme4). The model was fitted by specifying the fixed effects: treatment (categorical predictor) and the random effect: Plasmodium. N> 50 replicates for each treatment. $N_{Plasmodium}$ Plasmodium Identity.

	Response Variable: Sigma parameter, vein length				
Predictors (Explanatory variables)	*Estimates*	*CI*	*Statistic*	*p*	*df*
Intercept	-0.12	-0.42 – 0.17	-0.82	0.412	148.00
[Neutral] vs [Nutritive]	0.50	0.14 – 0.86	2.74	**0.006**	148.00
[Neutral] vs [Adverse]	-0.33	-0.72 – 0.07	-1.64	0.102	148.00
**Random Effects**					
Within-group variance σ^2	0.85				
Between-group-variance $\tau_{00\ Plasmodium}$	0.03				
Intraclass-Correlation Coefficient	0.04				
$N_{Plasmodium}$	12				
Observations	153				
Marginal R^2 / Conditional R^2	0.125 / 0.158				

Table S15: sigma parameter, vein length.
To assess the difference in sigma parameter extracted from the log-logistic fit of the vein length distribution between the three treatments; we used a generalized linear mixed model (function glmer, Package lme4). The model was fitted by specifying the fixed effects: treatment (categorical predictor) and the random effect: Plasmodium. N> 50 replicates for each treatment. $N_{Plasmodium}$ Plasmodium Identity.

	Response Variable: Tortuosity				
Predictors (Explanatory variables)	*Estimates*	*CI*	*Statistic*	*p*	*df*
Intercept	-0.17	-0.50 – 0.15	-1.04	0.298	148.00
[Neutral] vs [Nutritive]	0.38	0.01 – 0.76	2.01	**0.044**	148.00
[Neutral] vs [Adverse]	-0.09	-0.50 – 0.32	-0.42	0.674	148.00
**Random Effects**					
Within-group variance σ^2	0.90				
Between-group-variance $\tau_{00\ Plasmodium}$	0.08				
Intraclass-Correlation Coefficient	0.08				
$N_{Plasmodium}$	12				
Observations	153				
Marginal R^2 / Conditional R^2	0.045 / 0.119				

Table S16: Tortuosity Statistics associated to Figure 9a.
To assess the difference in tortuosity between the three treatments; we used a generalized linear mixed model (function glmer, Package lme4). The model was fitted by specifying the fixed effects: treatment (categorical predictor) and the random effect: Plasmodium. N> 50 replicates for each treatment. $N_{Plasmodium}$ Plasmodium Identity.

	Response Variable: Normalized Network Length				
Predictors (Explanatory variables)	*Estimates*	*CI*	*Statistic*	*p*	*df*
Intercept	0.44	0.08 – 0.80	2.42	**0.016**	148.00
[Neutral] vs [Nutritive]	-0.51	-0.85 – -0.16	-2.89	**0.004**	148.00
[Neutral] vs [Adverse]	-0.36	-0.74 – 0.01	-1.89	0.059	148.00
**Random Effects**					
Within-group variance σ^2	0.74				
Between-group-variance $\tau_{00\ Plasmodium}$	0.18				
Intraclass-Correlation Coefficient	0.20				
$N_{Plasmodium}$	12				
Observations	153				
Marginal R^2 / Conditional R^2	0.046 / 0.235				

Table S17: Normalized Network Length Statistics associated to Figure 9b.
To assess the difference in Normalized Network Length between the three treatments; we used a generalized linear mixed model (function glmer, Package lme4). The model was fitted by specifying the fixed effects: treatment (categorical predictor) and the random effect: Plasmodium. N> 50 replicates for each treatment. $N_{Plasmodium}$ Plasmodium Identity.

	Response Variable: Length efficiency (mean)				
Predictors (Explanatory variables)	*Estimates*	*CI*	*Statistic*	*p*	*df*
Intercept	0.27	-0.05 – 0.59	1.65	0.099	148.00
[Neutral] vs [Nutritive]	-0.70	-1.02 – -0.38	-4.26	**<0.001**	148.00
[Neutral] vs [Adverse]	0.31	-0.04 – 0.66	1.74	0.082	148.00
**Random Effects**					
Within-group variance σ^2	0.66				
Between-group-variance $\tau_{00\ Plasmodium}$	0.13				
Intraclass-Correlation Coefficient	0.16				
$N_{Plasmodium}$	12				
Observations	153				
Marginal R^2 / Conditional R^2	0.200 / 0.332				

Table S18: Length efficiency (mean) Statistics associated to Figure 10a.
To assess the difference in length efficiency (mean) between the three treatments; we used a generalized linear mixed model (function glmer, Package lme4). The model was fitted by specifying the fixed effects: treatment (categorical predictor) and the random effect: Plasmodium. N> 50 replicates for each treatment. $N_{Plasmodium}$ Plasmodium Identity.

	Response Variable: Length efficiency (coefficient of variation)				
Predictors (Explanatory variables)	*Estimates*	*CI*	*Statistic*	*p*	*df*
Intercept	-0.52	-0.89 – -0.14	-2.71	**0.007**	148.00
[Neutral] vs [Nutritive]	0.76	0.46 – 1.05	5.05	**<0.001**	148.00
[Neutral] vs [Adverse]	-0.05	-0.38 – 0.27	-0.34	0.737	148.00
**Random Effects**					
Within-group variance σ^2	0.53				
Between-group-variance $\tau_{00\ Plasmodium}$	0.28				
Intraclass-Correlation Coefficient	0.34				
$N_{Plasmodium}$	12				
Observations	153				
Marginal R^2 / Conditional R^2	0.159 / 0.447				

Table S19: Length efficiency (coefficient of variation) Statistics associated to Figure 10a.
To assess the difference in length efficiency (coefficient of variation) between the three treatments; we used a generalized linear mixed model (function glmer, Package lme4). The model was fitted by specifying the fixed effects: treatment (categorical predictor) and the random effect: Plasmodium. N> 50 replicates for each treatment. $N_{Plasmodium}$ Plasmodium Identity.

	Response Variable: Drag efficiency (mean)				
Predictors (Explanatory variables)	*Estimates*	*CI*	*Statistic*	*p*	*df*
Intercept	-1.16	-1.49 – -0.84	-7.02	**<0.001**	148.00
[Neutral] vs [Nutritive]	1.21	0.96 – 1.46	9.55	**<0.001**	148.00
[Neutral] vs [Adverse]	1.23	0.96 – 1.50	8.88	**<0.001**	148.00
**Random Effects**					
Within-group variance σ^2	0.38				
Between-group-variance $\tau_{00\ Plasmodium}$	0.22				
Intraclass-Correlation Coefficient	0.36				
$N_{Plasmodium}$	12				
Observations	153				
Marginal R^2 / Conditional R^2	0.336 / 0.576				

Table S20: Drag efficiency (mean) Statistics associated to Figure 10b.
To assess the difference in Drag efficiency (mean) between the three treatments; we used a generalized linear mixed model (function glmer, Package lme4). The model was fitted by specifying the fixed effects: treatment (categorical predictor) and the random effect: Plasmodium. N> 50 replicates for each treatment. $N_{Plasmodium}$ Plasmodium Identity.

	**Response Variable: Drag efficiency (coefficient of variation)**				
Predictors (Explanatory variables)	*Estimates*	*CI*	*Statistic*	*p*	*df*
Intercept	-0.32	-0.62 – -0.03	-2.13	**0.033**	148.00
[Neutral] vs [Nutritive]	0.48	0.10 – 0.86	2.47	**0.014**	148.00
[Neutral] vs [Adverse]	0.41	-0.00 – 0.82	1.94	0.052	148.00
**Random Effects**					
Within-group variance σ^2	0.97				
Between-group-variance $\tau_{00\ Plasmodium}$	0.00				
Intraclass-Correlation Coefficient	0.00				
$N_{Plasmodium}$	12				
Observations	153				
Marginal R^2 / Conditional R^2	0.041 / 0.044				

Table S21: Drag efficiency (coefficient of variation) Statistics associated to Figure 10b.
To assess the difference in Drag efficiency (coefficient of variation) between the three treatments; we used a generalized linear mixed model (function glmer, Package lme4). The model was fitted by specifying the fixed effects: treatment (categorical predictor) and the random effect: Plasmodium. N> 50 replicates for each treatment. $N_{Plasmodium}$ Plasmodium Identity.

	**Response Variable: Edge betweenness distribution by length**				
Predictors (Explanatory variables)	*Estimates*	*CI*	*Statistic*	*p*	*df*
Intercept	-0.71	-1.12 – -0.29	-3.35	**0.001**	148.00
[Neutral] vs [Nutritive]	0.51	0.22 – 0.80	3.49	**<0.001**	148.00
[Neutral] vs [Adverse]	0.36	0.04 – 0.67	2.23	**0.025**	148.00
**Random Effects**					
Within-group variance σ^2	0.50				
Between-group-variance $\tau_{00\ Plasmodium}$	0.38				
Intraclass-Correlation Coefficient	0.43				
$N_{Plasmodium}$	12				
Observations	153				
Marginal R^2 / Conditional R^2	0.049 / 0.458				

Table S22: Edge betweenness distribution by length Statistics associated to Figure 11a.
To assess the difference in Edge betweenness distribution by length between the three treatments; we used a generalized linear mixed model (function glmer, Package lme4). The model was fitted by specifying the fixed effects: treatment (categorical predictor) and the random effect: Plasmodium. N> 50 replicates for each treatment. $N_{Plasmodium}$ Plasmodium Identity.

	**Response Variable: Edge betweenness distribution by drag**				
Predictors (Explanatory variables)	*Estimates*	*CI*	*Statistic*	*p*	*df*
Intercept	-0.73	-1.15 – -0.31	-3.43	**0.001**	148.00
[Neutral] vs [Nutritive]	0.58	0.30 – 0.85	4.11	**<0.001**	148.00
[Neutral] vs [Adverse]	0.29	-0.01 – 0.59	1.88	0.061	148.00
**Random Effects**					
Within-group variance σ^2	0.46				
Between-group-variance $\tau_{00\ Plasmodium}$	0.41				
Intraclass-Correlation Coefficient	0.47				
$N_{Plasmodium}$	12				
Observations	153				
Marginal R^2 / Conditional R^2	0.062 / 0.503				

Table S23: Edge betweenness distribution by drag Statistics associated to Figure 11b.
To assess the difference in Edge betweenness distribution by drag between the three treatments; we used a generalized linear mixed model (function glmer, Package lme4). The model was fitted by specifying the fixed effects: treatment (categorical predictor) and the random effect: Plasmodium. N> 50 replicates for each treatment. $N_{Plasmodium}$ Plasmodium Identity.

	Response Variable: Normalized fault tolerances 25				
Predictors (Explanatory variables)	*Estimates*	*CI*	*Statistic*	*p*	*df*
Intercept	0.32	-0.03 – 0.67	1.80	0.072	148.00
[Neutral] vs [Nutritive]	-0.66	-0.99 – -0.33	-3.95	**<0.001**	148.00
[Neutral] vs [Adverse]	0.08	-0.28 – 0.44	0.45	0.655	148.00
**Random Effects**					
Within-group variance σ^2	0.67				
Between-group-variance $\tau_{00\ Plasmodium}$	0.18				
Intraclass-Correlation Coefficient	0.21				
$N_{Plasmodium}$	12				
Observations	153				
Marginal R^2 / Conditional R^2	0.126 / 0.313				

Table S24: Normalized fault tolerances 25 Statistics associated to Figure 12.
To assess the difference in Normalized fault tolerances 25 between the three treatments; we used a generalized linear mixed model (function glmer, Package lme4). The model was fitted by specifying the fixed effects: treatment (categorical predictor) and the random effect: Plasmodium. N> 50 replicates for each treatment. $N_{Plasmodium}$ Plasmodium Identity.

	Response Variable: Normalized fault tolerances 50				
Predictors (Explanatory variables)	*Estimates*	*CI*	*Statistic*	*p*	*df*
Intercept	0.48	0.07 – 0.88	2.31	**0.021**	148.00
[Neutral] vs [Nutritive]	-0.76	-1.06 – -0.46	-4.95	**<0.001**	148.00
[Neutral] vs [Adverse]	-0.27	-0.60 – 0.06	-1.61	0.107	148.00
**Random Effects**					
Within-group variance σ^2	0.56				
Between-group-variance $\tau_{00\ Plasmodium}$	0.34				
Intraclass-Correlation Coefficient	0.38				
$N_{Plasmodium}$	12				
Observations	153				
Marginal R^2 / Conditional R^2	0.106 / 0.446				

Table S25: Normalized fault tolerances 50 Statistics associated to Figure 12.
To assess the difference in Normalized fault tolerances 50 between the three treatments; we used a generalized linear mixed model (function glmer, Package lme4). The model was fitted by specifying the fixed effects: treatment (categorical predictor) and the random effect: Plasmodium. N> 50 replicates for each treatment. $N_{Plasmodium}$ Plasmodium Identity.

	Response Variable: Normalized fault tolerances 75				
Predictors (Explanatory variables)	*Estimates*	*CI*	*Statistic*	*p*	*df*
Intercept	0.50	0.11 – 0.88	2.55	**0.011**	148.00
[Neutral] vs [Nutritive]	-0.82	-1.11 – -0.53	-5.50	**<0.001**	148.00
[Neutral] vs [Adverse]	-0.16	-0.48 – 0.16	-1.00	0.316	148.00
**Random Effects**					
Within-group variance σ^2	0.53				
Between-group-variance $\tau_{00\ Plasmodium}$	0.30				
Intraclass-Correlation Coefficient	0.36				
$N_{Plasmodium}$	12				
Observations	153				
Marginal R^2 / Conditional R^2	0.143 / 0.456				

Table S26: Normalized fault tolerances 75 Statistics associated to Figure 12.
To assess the difference in Normalized fault tolerances 75 between the three treatments; we used a generalized linear mixed model (function glmer, Package lme4). The model was fitted by specifying the fixed effects: treatment (categorical predictor) and the random effect: Plasmodium. N> 50 replicates for each treatment. $N_{Plasmodium}$ Plasmodium Identity.

	Response Variable: Normalized fault tolerances				
Predictors (Explanatory variables)	*Estimates*	*CI*	*Statistic*	*p*	*df*
Intercept	0.15	0.05 – 0.25	3.02	**0.003**	145.00
[Neutral] vs [Nutritive]	-0.10	-0.21 – 0.01	-1.84	0.066	145.00
[Neutral] vs [Adverse]	0.08	-0.06 – 0.21	1.11	0.269	145.00
Normalized Network Length [NNL]	1.21	0.62 – 1.79	4.07	**<0.001**	145.00
[Neutral]*[NNL] vs [Glucose]*[NNL]	0.43	-0.21 – 1.07	1.33	0.185	145.00
[Neutral]*[NNL] vs [NaCl]*[NNL]	-0.45	-1.27 – 0.37	-1.08	0.279	145.00
**Random Effects**					
Within-group variance σ^2	0.00				
Between-group-variance $\tau_{00\ Plasmodium}$	0.00				
Intraclass-Correlation Coefficient	0.13				
$N_{Plasmodium}$	12				
Observations	153				
Marginal R^2 / Conditional R^2	0.569 / 0.623				

Table S27: Normalized fault tolerances as a function of Normalized network length. Statistics associated to Figure 13a. To assess the difference in Normalized fault tolerances between the three treatments as a function of Normalized network length; we used a linear mixed model (function lmer, Package lme4). The model was fitted by specifying the fixed effects: treatment (categorical predictor) and Normalized network length (continuous predictor) and the random effects: Plasmodium and Replicate. N> 50 replicates for each treatment. $N_{Plasmodium}$ Plasmodium Identity.

	Response Variable: Edge betweenness distribution by drag				
Predictors (Explanatory variables)	*Estimates*	*CI*	*Statistic*	*p*	*df*
Intercept	0.09	0.06 – 0.12	6.16	**<0.001**	145.00
[Neutral] vs [Nutritive]	0.02	-0.01 – 0.05	1.24	0.216	145.00
[Neutral] vs [Adverse]	-0.00	-0.04 – 0.03	-0.14	0.887	145.00
Normalized Network Length [NNL]	-0.32	-0.48 – -0.15	-3.78	**<0.001**	145.00
[Neutral]*[NNL] vs [Glucose]*[NNL]	-0.08	-0.26 – 0.10	-0.91	0.362	145.00
[Neutral]*[NNL] vs [NaCl]*[NNL]	0.03	-0.20 – 0.25	0.23	0.816	145.00
**Random Effects**					
Within-group variance σ^2	0.00				
Between-group-variance $\tau_{00\ Plasmodium}$	0.00				
Intraclass-Correlation Coefficient	0.38				
$N_{Plasmodium}$	12				
Observations	153				
Marginal R^2 / Conditional R^2	0.410 / 0.633				

Table S28: Edge betweenness distribution by drag as a function of Normalized network length. Statistics associated to Figure 13b.
To assess the difference in Edge betweenness distribution by drag between the three treatments as a function of Normalized network length; we used a linear mixed model (function lmer, Package lme4). The model was fitted by specifying the fixed effects: treatment (categorical predictor) and Normalized network length (continuous predictor) and the random effects: Plasmodium and Replicate. N> 50 replicates for each treatment. $N_{Plasmodium}$ Plasmodium Identity.

	Response Variable: Normalized slime mold fusion area				
Predictors (Explanatory variables)	*Estimates*	*CI*	*Statistic*	*p*	*df*
Intercept	-0.02	-0.37 – 0.34	-0.10	0.921	1044.00
[Neutral] vs [Nutritive]	0.30	-0.04 – 0.65	1.71	0.087	1044.00
[Neutral] vs [Adverse]	0.53	0.14 – 0.92	2.66	**0.008**	1044.00
Time [t]	-0.01	-0.01 – -0.01	-12.19	**<0.001**	1044.00
[t]:[Neutral] vs [t]:[Nutritive]	0.00	0.00 – 0.00	2.63	**0.009**	1044.00
[t]:[Neutral] vs [t]:[Adverse]	0.01	0.00 – 0.01	6.54	**<0.001**	1044.00
**Random Effects**					
Within-group variance σ^2	0.33				
Between-group-variance $\tau_{00\ ID:Plasmodium}$	0.35				
Between-group-variance $\tau_{00\ Plasmodium}$	0.16				
Intraclass-Correlation Coefficient	0.60				
N_{ID}	81				
$N_{Plasmodium}$	12				
Observations	1053				
Marginal R^2 / Conditional R^2	0.217 / 0.691				

Table S29: Normalized slime mold fusion area Statistics associated to Figure 17a.
To assess the difference in Normalized slime mold fusion area between the three treatments; we used a linear mixed model (function lmer, Package lme4). The model was fitted by specifying the fixed effects: treatment (categorical predictor) and time from fusion (continuous predictor) and the random effects: Plasmodium and Replicate. The dependent variable (Normalized slime mold fusion area) was normalized using the function bestNormalize (Package bestNormalize). N> 25 replicates for each treatment. $N_{Plasmodium}$ Plasmodium Identity.

	Response Variable: Normalized print area				
Predictors (Explanatory variables)	*Estimates*	*CI*	*Statistic*	*p*	*df*
Intercept	-0.17	-0.53 – 0.19	-0.94	0.347	1044.00
[Neutral] vs [Nutritive]	0.02	-0.37 – 0.40	0.08	0.937	1044.00
[Neutral] vs [Adverse]	0.24	-0.19 – 0.66	1.09	0.276	1044.00
Time [t]	-0.00	-0.00 – -0.00	-5.46	**<0.001**	1044.00
[t]:[Neutral] vs [t]:[Nutritive]	0.00	0.00 – 0.01	5.01	**<0.001**	1044.00
[t]:[Neutral] vs [t]:[Adverse]	0.00	0.00 – 0.01	4.72	**<0.001**	1044.00
**Random Effects**					
Within-group variance σ^2	0.44				
Between-group-variance $\tau_{00\ ID:Plasmodium}$	0.41				
Between-group-variance $\tau_{00\ Plasmodium}$	0.12				
Intraclass-Correlation Coefficient	0.54				
N_{ID}	81				
$N_{Plasmodium}$	12				
Observations	1053				
Marginal R^2 / Conditional R^2	0.072 / 0.578				

Table S30: Normalized print area Statistics associated to Figure 17b.
To assess the difference in Normalized print area between the three treatments; we used a linear mixed model (function lmer, Package lme4). The model was fitted by specifying the fixed effects: treatment (categorical predictor) and time from fusion (continuous predictor) and the random effects: Plasmodium and Replicate. The dependent variable (Normalized print area) was normalized using the function bestNormalize (Package bestNormalize). N> 25 replicates for each treatment. $N_{Plasmodium}$ Plasmodium Identity.

	Response Variable: Empty area – Slime mold ratio				
Predictors (Explanatory variables)	*Estimates*	*CI*	*Statistic*	*p*	*df*
Intercept	-0.49	-0.82 – -0.17	-2.97	**0.003**	1044.00
[Neutral] vs [Nutritive]	0.16	-0.26 – 0.58	0.75	0.456	1044.00
[Neutral] vs [Adverse]	-0.07	-0.54 – 0.40	-0.28	0.781	1044.00
Time [t]	0.00	0.00 – 0.00	6.60	**<0.001**	1044.00
[t]:[Neutral] vs [t]:[Nutritive]	0.00	0.00 – 0.00	4.43	**<0.001**	1044.00
[t]:[Neutral] vs [t]:[Adverse]	-0.00	-0.00 – 0.00	-0.58	0.559	1044.00
**Random Effects**					
Within-group variance σ^2	0.30				
Between-group-variance $\tau_{00\ ID:Plasmodium}$	0.57				
Between-group-variance $\tau_{00\ Plasmodium}$	0.00				
Intraclass-Correlation Coefficient	0.66				
N_{ID}	81				
$N_{Plasmodium}$	12				
Observations	1053				
Marginal R^2 / Conditional R^2	0.144 / 0.706				

Table S31: Empty area – Slime mold ratio Statistics associated to Figure 17c.
To assess the difference in Empty area – Slime mold ratio between the three treatments; we used a linear mixed model (function lmer, Package lme4). The model was fitted by specifying the fixed effects: treatment (categorical predictor) and time from fusion (continuous predictor) and the random effects: Plasmodium and Replicate. The dependent variable (Empty area – Slime mold ratio) was normalized using the function bestNormalize (Package bestNormalize). N> 25 replicates for each treatment. $N_{Plasmodium}$ Plasmodium Identity.

	Response Variable: Normalized network length				
Predictors (Explanatory variables)	*Estimates*	*CI*	*Statistic*	*p*	*df*
Intercept	-0.28	-0.66 – 0.09	-1.48	0.140	1044.00
[Neutral] vs [Nutritive]	-0.05	-0.46 – 0.36	-0.25	0.800	1044.00
[Neutral] vs [Adverse]	0.08	-0.37 – 0.54	0.36	0.716	1044.00
Time [t]	-0.00	-0.00 – 0.00	-0.33	0.738	1044.00
[t]:[Neutral] vs [t]:[Nutritive]	0.00	0.00 – 0.00	3.82	**<0.001**	1044.00
[t]:[Neutral] vs [t]:[Adverse]	0.00	-0.00 – 0.00	1.60	0.109	1044.00
**Random Effects**					
Within-group variance σ^2	0.40				
Between-group-variance $\tau_{00\ ID:Plasmodium}$	0.50				
Between-group-variance $\tau_{00\ Plasmodium}$	0.12				
Intraclass-Correlation Coefficient	0.61				
N_{ID}	81				
$N_{Plasmodium}$	12				
Observations	1053				
Marginal R^2 / Conditional R^2	0.023 / 0.615				

Table S32: Normalized network length Statistics associated to Figure 18a.
To assess the difference in Normalized network lenght between the three treatments; we used a linear mixed model (function lmer, Package lme4). The model was fitted by specifying the fixed effects: treatment (categorical predictor) and time from fusion (continuous predictor) and the random effects: Plasmodium and Replicate. The dependent variable (Normalized network lenght) was normalized using the function bestNormalize (Package bestNormalize). N> 25 replicates for each treatment. $N_{Plasmodium}$ Plasmodium Identity.

	**Response Variable: Average vein width**				
Predictors (Explanatory variables)	*Estimates*	*CI*	*Statistic*	*p*	*df*
Intercept	0.13	-0.15 – 0.41	0.93	0.350	1044.00
[Neutral] vs [Nutritive]	0.44	0.13 – 0.76	2.75	**0.006**	1044.00
[Neutral] vs [Adverse]	0.81	0.46 – 1.16	4.51	**<0.001**	1044.00
Time [t]	-0.01	-0.01 – -0.01	-14.75	**<0.001**	1044.00
[t]:[Neutral] vs [t]:[Nutritive]	-0.00	-0.00 – 0.00	-1.82	0.069	1044.00
[t]:[Neutral] vs [t]:[Adverse]	0.00	0.00 – 0.01	5.59	**<0.001**	1044.00
**Random Effects**					
Within-group variance σ^2	0.27				
Between-group-variance $\tau_{00\ ID:Plasmodium}$	0.29				
Between-group-variance $\tau_{00\ Plasmodium}$	0.05				
Intraclass-Correlation Coefficient	0.55				
N_{ID}	81				
$N_{Plasmodium}$	12				
Observations	1053				
Marginal R^2 / Conditional R^2	0.391 / 0.728				

Table S33: Average vein width Statistics associated to Figure 18b.
To assess the difference in Average vein width between the three treatments; we used a linear mixed model (function lmer, Package lme4). The model was fitted by specifying the fixed effects: treatment (categorical predictor) and time from fusion (continuous predictor) and the random effects: Plasmodium and Replicate. The dependent variable (Average vein width) was normalized using the function bestNormalize (Package bestNormalize). N> 25 replicates for each treatment. $N_{Plasmodium}$ Plasmodium Identity.

	**Response Variable: Maximum vein width**				
Predictors (Explanatory variables)	*Estimates*	*CI*	*Statistic*	*p*	*df*
Intercept	0.17	-0.09 – 0.44	1.27	0.206	1044.00
[Neutral] vs [Nutritive]	0.59	0.30 – 0.88	3.98	**<0.001**	1044.00
[Neutral] vs [Adverse]	0.60	0.27 – 0.92	3.63	**<0.001**	1044.00
Time [t]	-0.01	-0.01 – -0.01	-16.48	**<0.001**	1044.00
[t]:[Neutral] vs [t]:[Nutritive]	0.00	0.00 – 0.01	4.49	**<0.001**	1044.00
[t]:[Neutral] vs [t]:[Adverse]	0.01	0.00 – 0.01	7.17	**<0.001**	1044.00
**Random Effects**					
Within-group variance σ^2	0.36				
Between-group-variance $\tau_{00\ ID:Plasmodium}$	0.21				
Between-group-variance $\tau_{00\ Plasmodium}$	0.06				
Intraclass-Correlation Coefficient	0.43				
N_{ID}	81				
$N_{Plasmodium}$	12				
Observations	1053				
Marginal R^2 / Conditional R^2	0.378 / 0.644				

Table S34: Maximum vein width Statistics associated to Figure 18c.
To assess the difference in Maximum vein width between the three treatments; we used a linear mixed model (function lmer, Package lme4). The model was fitted by specifying the fixed effects: treatment (categorical predictor) and time from fusion (continuous predictor) and the random effects: Plasmodium and Replicate. The dependent variable (Maximum vein width) was normalized using the function bestNormalize (Package bestNormalize). N> 25 replicates for each treatment. $N_{Plasmodium}$ Plasmodium Identity.

	Response Variable: Number of connecting veins				
Predictors (Explanatory variables)	*Incidence Rate Ratios*	*CI*	*Statistic*	*p*	*df*
Intercept	2.00	1.56 – 2.57	5.50	**<0.001**	1045.00
[Neutral] vs [Nutritive]	0.97	0.73 – 1.29	-0.18	0.858	1045.00
[Neutral] vs [Adverse]	0.82	0.59 – 1.14	-1.19	0.234	1045.00
Time [t]	1.01	1.01 – 1.01	12.48	**<0.001**	1045.00
[t]:[Neutral] vs [t]:[Nutritive]	1.00	1.00 – 1.00	-1.93	0.054	1045.00
[t]:[Neutral] vs [t]:[Adverse]	0.99	0.99 – 1.00	-5.74	**<0.001**	1045.00
**Random Effects**					
Within-group variance σ^2	0.30				
Between-group-variance $\tau_{00\ ID:Plasmodium}$	0.19				
Between-group-variance $\tau_{00\ Plasmodium}$	0.04				
Intraclass-Correlation Coefficient	0.43				
N_{ID}	81				
$N_{Plasmodium}$	12				
Observations	1053				
Marginal R^2 / Conditional R^2	0.218 / 0.555				

Table S35: Number of connecting veins Statistics associated to Figure 19a.
To assess the difference in the Number of connecting veins between the three treatments; we used a generalized linear mixed model (function glmer, Package lme4). The model was fitted by specifying the fixed effects: treatment (categorical predictor) and time from fusion (continuous predictor) and the random effects: Plasmodium and Replicate. N> 25 replicates for each treatment. $N_{Plasmodium}$ Plasmodium Identity.

	Response Variable: Connecting vein width				
Predictors (Explanatory variables)	*Estimates*	*CI*	*Statistic*	*p*	*df*
Intercept	-0.31	-0.62 – -0.01	-2.03	**0.042**	1035.00
[Neutral] vs [Nutritive]	0.31	-0.02 – 0.64	1.81	0.070	1035.00
[Neutral] vs [Adverse]	0.59	0.22 – 0.96	3.11	**0.002**	1035.00
Time [t]	-0.00	-0.00 – 0.00	-1.78	0.076	1035.00
[t]:[Neutral] vs [t]:[Nutritive]	-0.00	-0.00 – 0.00	-0.01	0.991	1035.00
[t]:[Neutral] vs [t]:[Adverse]	0.00	-0.00 – 0.00	0.31	0.758	1035.00
**Random Effects**					
Within-group variance σ^2	0.66				
Between-group-variance $\tau_{00\ ID:Plasmodium}$	0.22				
Between-group-variance $\tau_{00\ Plasmodium}$	0.07				
Intraclass-Correlation Coefficient	0.31				
N_{ID}	81				
$N_{Plasmodium}$	12				
Observations	1044				
Marginal R^2 / Conditional R^2	0.060 / 0.352				

Table S36: Connecting vein width Statistics associated to Figure 19b.
To assess the difference in Connecting vein width between the three treatments; we used a linear mixed model (function lmer, Package lme4). The model was fitted by specifying the fixed effects: treatment (categorical predictor) and time from fusion (continuous predictor) and the random effects: Plasmodium and Replicate. The dependent variable (Connecting vein width) was normalized using the function bestNormalize (Package bestNormalize). N> 25 replicates for each treatment. $N_{Plasmodium}$ Plasmodium Identity.

	Response Variable: Path length between cells				
Predictors (Explanatory variables)	*Estimates*	*CI*	*Statistic*	*p*	*df*
Intercept	-0.30	-0.66 – 0.07	-1.59	0.113	1044.00
[Neutral] vs [Nutritive]	0.26	-0.14 – 0.66	1.29	0.199	1044.00
[Neutral] vs [Adverse]	0.74	0.29 – 1.19	3.23	**0.001**	1044.00
Time [t]	-0.00	-0.00 – -0.00	-3.06	**0.002**	1044.00
[t]:[Neutral] vs [t]:[Nutritive]	0.00	-0.00 – 0.00	0.56	0.577	1044.00
[t]:[Neutral] vs [t]:[Adverse]	0.00	0.00 – 0.00	2.88	**0.004**	1044.00
**Random Effects**					
Within-group variance σ^2	0.30				
Between-group-variance $\tau_{00\ ID:Plasmodium}$	0.50				
Between-group-variance $\tau_{00\ Plasmodium}$	0.11				
Intraclass-Correlation Coefficient	0.67				
N_{ID}	81				
$N_{Plasmodium}$	12				
Observations	1053				
Marginal R^2 / Conditional R^2	0.132 / 0.715				

Table S37: Path length between cells Statistics associated to Figure 19c.
To assess the difference in Path length between cells between the three treatments; we used a linear mixed model (function lmer, Package lme4). The model was fitted by specifying the fixed effects: treatment (categorical predictor) and time from fusion (continuous predictor) and the random effects: Plasmodium and Replicate. The dependent variable (Path length between cells) was normalized using the function bestNormalize (Package bestNormalize). N> 25 replicates for each treatment. $N_{Plasmodium}$ Plasmodium Identity.

	Response Variable: Path drag between cells				
Predictors (Explanatory variables)	*Estimates*	*CI*	*Statistic*	*p*	*df*
Intercept	1.14	0.80 – 1.48	6.57	**<0.001**	1044.00
[Neutral] vs [Nutritive]	-1.12	-1.45 – -0.79	-6.65	**<0.001**	1044.00
[Neutral] vs [Adverse]	-1.05	-1.41 – -0.68	-5.55	**<0.001**	1044.00
Time [t]	-0.00	-0.00 – -0.00	-4.52	**<0.001**	1044.00
[t]:[Neutral] vs [t]:[Nutritive]	0.00	0.00 – 0.00	2.62	**0.009**	1044.00
[t]:[Neutral] vs [t]:[Adverse]	-0.00	-0.00 – 0.00	-1.14	0.253	1044.00
**Random Effects**					
Within-group variance σ^2	0.32				
Between-group-variance $\tau_{00\ ID:Plasmodium}$	0.31				
Between-group-variance $\tau_{00\ Plasmodium}$	0.15				
Intraclass-Correlation Coefficient	0.59				
N_{ID}	81				
$N_{Plasmodium}$	12				
Observations	1053				
Marginal R^2 / Conditional R^2	0.235 / 0.688				

Table S38: Path drag between cells Statistics associated to Figure 19d.
To assess the difference in Path drag between cells between the three treatments; we used a linear mixed model (function lmer, Package lme4). The model was fitted by specifying the fixed effects: treatment (categorical predictor) and time from fusion (continuous predictor) and the random effects: Plasmodium and Replicate. The dependent variable (Path drag between cells) was normalized using the function bestNormalize (Package bestNormalize). N> 25 replicates for each treatment. $N_{Plasmodium}$ Plasmodium Identity.

Supplementary Figures

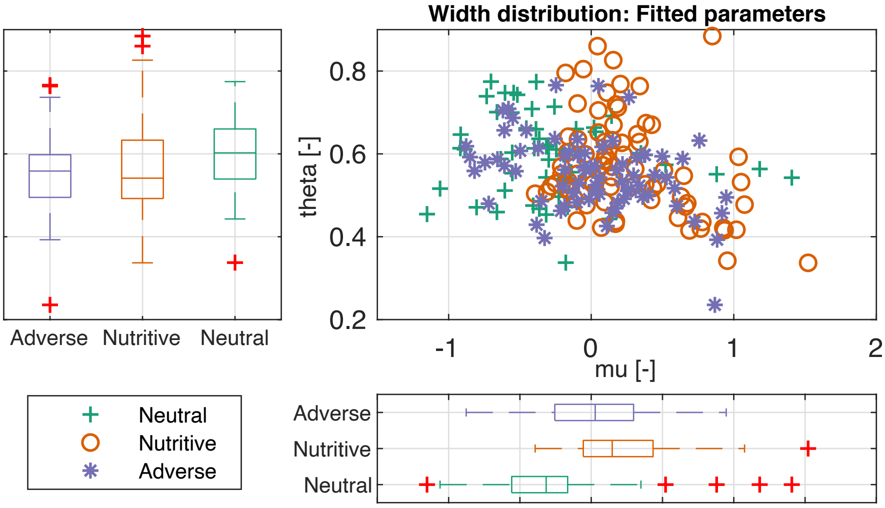

Figure S39: Vein width distribution - fitted parameters.
The vein width frequency distribution has been fitted using a loglogistic regression and its parameters: theta – shape, and mu – location (mean) plotted for the different environments.

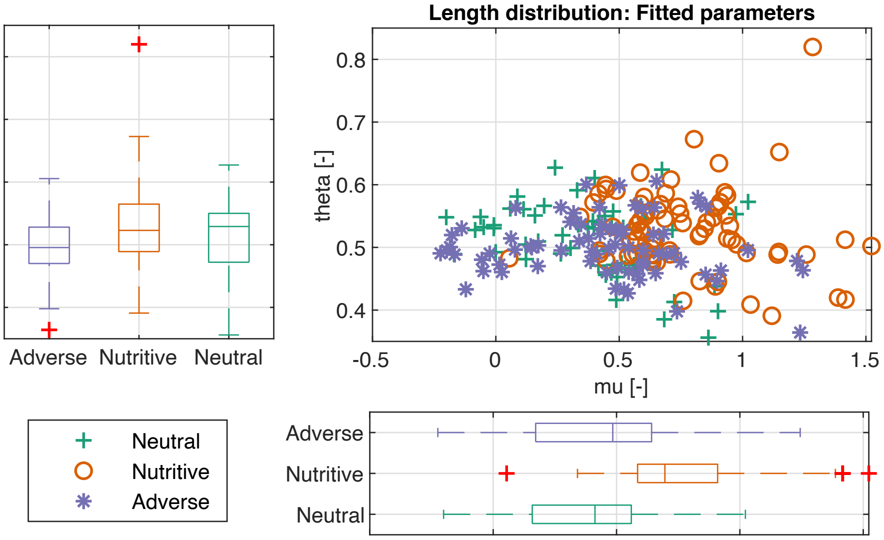

Figure S40: Vein length distribution - fitted parameters.
The vein length frequency distribution has been fitted using a loglogistic regression and its parameters: theta – shape, and mu – location (mean) plotted for the different environments.
